# Phenolic-Rich Plant Extracts With Antimicrobial Activity: An Alternative to Food Preservatives and Biocides?

**DOI:** 10.3389/fmicb.2021.753518

**Published:** 2022-01-04

**Authors:** Nadia Oulahal, Pascal Degraeve

**Affiliations:** Univ Lyon, Université Claude Bernard Lyon 1, ISARA Lyon, BioDyMIA (Bioingénierie et Dynamique Microbienne aux Interfaces Alimentaires), Equipe Mixte d’Accueil n°3733, IUT Lyon 1, Technopole Alimentec, Bourg-en-Bresse, France

**Keywords:** phenolic-rich plant extracts, antimicrobial activity, food preservation, biocides, biofilms, delivery systems

## Abstract

In recent years, the search for natural plant-based antimicrobial compounds as alternatives to some synthetic food preservatives or biocides has been stimulated by sanitary, environmental, regulatory, and marketing concerns. In this context, besides their established antioxidant activity, the antimicrobial activity of many plant phenolics deserved increased attention. Indeed, industries processing agricultural plants generate considerable quantities of phenolic-rich products and by-products, which could be valuable natural sources of natural antimicrobial molecules. Plant extracts containing volatile (e.g., essential oils) and non-volatile antimicrobial molecules can be distinguished. Plant essential oils are outside the scope of this review. This review will thus provide an overview of current knowledge regarding the promises and the limits of phenolic-rich plant extracts for food preservation and biofilm control on food-contacting surfaces. After a presentation of the major groups of antimicrobial plant phenolics, of their antimicrobial activity spectrum, and of the diversity of their mechanisms of action, their most promising sources will be reviewed. Since antimicrobial activity reduction often observed when comparing *in vitro* and *in situ* activities of plant phenolics has often been reported as a limit for their application, the effects of the composition and the microstructure of the matrices in which unwanted microorganisms are present (e.g., food and/or microbial biofilms) on their activity will be discussed. Then, the different strategies of delivery of antimicrobial phenolics to promote their activity in such matrices, such as their encapsulation or their association with edible coatings or food packaging materials are presented. The possibilities offered by encapsulation or association with polymers of packaging materials or coatings to increase the stability and ease of use of plant phenolics before their application, as well as to get systems for their controlled release are presented and discussed. Finally, the necessity to consider phenolic-rich antimicrobial plant extracts in combination with other factors consistently with hurdle technology principles will be discussed. For instance, several authors recently suggested that natural phenolic-rich extracts could not only extend the shelf-life of foods by controlling bacterial contamination, but could also coexist with probiotic lactic acid bacteria in food systems to provide enhanced health benefits to human.

## Introduction

Search for natural alternatives to synthetic food preservatives and disinfectants has been the subject of intensive research during the last decade. It has namely been stimulated by increasing concerns regarding their innocuity [e.g., nitrites (EFSA Panel et al., 2017), sulfites (EFSA Panel, 2016), polyhexamethylenebiguanide (PHMB), 5-chloro-2-(2,4-dichlorophenoxy)phenol (triclosan; Müller and Kramer, 2008)] or their environmental impact [e.g., triclosan (Abbott et al., 2020)]. Regulatory changes [e.g., in the European Union (EU)] disinfectants are covered by the European Regulation concerning the marketing and use of biocidal products [Regulation (EU) no 528/2012, 2012] and increasing demand of consumers for organic foods (in which only a limited number of food preservatives are authorized) or for «clean label» food products (without or with a limited number of food additives such as food preservatives) are important drivers of this trend. New natural antimicrobial extracts/molecules are expected (i) to preserve raw foods (e.g., raw fish or meat) or foods minimally processed to better preserve their organoleptic and nutritional properties and (ii) to reduce food waste by extending shelf life of highly perishable foods [one of the objectives of Sustainable Development Goal (SDG) to “End hunger, achieve food security and improved nutrition and promote sustainable agriculture” of United Nations in line with EU objective to reach a 50% food waste reduction by 2030].

In this context, plant extracts are promising sources of antimicrobial molecules. Plant antimicrobial molecules are grouped into different classes based on their chemical structure and properties: essential oils, phenolics, alkaloids, saponins, and peptides (Ferdes, 2018). The present review is focused on phenolics, which are the most numerous among secondary metabolites groups of plants. Plant secondary metabolites (also called phytochemicals) are biosynthesized by plants as a result of biotic (e.g., contamination by phytopathogenic microorganisms) and abiotic factors (e.g., UV light; Harborne, 2001). Some phytochemicals are thus toxic and only edible plants will thus be considered as potential sources of alternative to food preservatives. Another trait of some plant phenols and polyphenols is their biosynthesis in response to seasonal fluctuations in UV light for plant protection (Köhler et al., 2017). This variability of phenolics content of plants as a function of climatic conditions must be kept in mind, when the objective is to produce plant extracts with a standard antimicrobial activity resulting from a given content in antimicrobial phenolics.

As stated by Cheynier et al. (2013), an ever-increasing number of different plant phenolics, representing tens of thousands of different chemical structures, have been identified. Progresses both in analytical techniques, namely mass spectrometry techniques, and in extraction and separation techniques result in a better knowledge of the structural diversity of plant phenolics and of their distribution in plant tissues, as recently reviewed by Piccolella et al. (2019). Essential oil phenolics, which are volatile molecules generally extracted from plants by steam distillation, will be out of the scope of the present review, since their potential use as alternatives to food preservatives (Falleh et al., 2020), as well as their capacity to fight against biofilms (Nuta et al., 2021), have been recently reviewed. Despite their large structural diversity, most antimicrobial plant phenolics belong to six groups: flavonoids, phenolic acids, tannins, stilbenoids, quinones, and coumarins (Bouarab-Chibane et al., 2018a).

The antimicrobial activity of most plant phenolics has been investigated *in vitro* in microbiological media with a far more simple composition and microstructure than food matrices and against planktonic bacteria. Namely due to interactions of plant phenolics with other food constituents at the expense of their interactions with microorganisms, most antimicrobial plant phenolics have a far lower activity in food than *in vitro*. A major concern for hygienic safety of food production systems is the presence of microbial biofilms on the surfaces of food production equipment and facilities. Microorganisms in biofilms are embedded in a network of exopolymeric substances. This network of exopolymeric substances limits direct contact of microorganisms with antimicrobial substances, including plant phenolics, exerting thereby a protective effect. Moreover, microorganisms in biofilms are in a particular physiological state, which also modifies their sensitivity to antimicrobial substances. Therefore, after a presentation of the structural diversity of plant phenolics and of their mechanisms of antimicrobial action, a focus on the factors limiting efficiency of antimicrobial plant phenolics in food and biofilms matrices, as well as on their specific mechanisms of action against biofilms, such as quorum sensing inhibition is proposed. Delivery vehicles promoting the antimicrobial activity of plant phenolics in food matrices or against biofilms (e.g., active coatings, films, or particles) are also presented.

## Structural Diversity of Antimicrobial Plant Phenolics

### Dietary Sources of Phenolics

Phenolics are a class of organic compounds that occurs in all plants as secondary metabolites in varying concentrations. Perez-Jimenez et al. (2010) exploited Phenol-Explorer database to identify the 100 richest dietary sources of polyphenols: they contain from 10 mg per 100 ml (rosé wine) to 15 g per 100 g (cloves). The richest sources are various spices and dried herbs, cocoa products, berries, some seeds, nuts, and some vegetables, including olive and globe artichoke heads. Due to their availability in sufficient amounts for a low cost, some by-products or wastes of phenolic-rich edible plants are promising sources of antimicrobial phenolics. The most studied sources in the 2007–2017 decade for extraction of antimicrobial phenolics were listed by Bouarab-Chibane et al. (2018a) and the potential of application of most of their extracts was recently reviewed by several authors: they include spent coffee (Monente et al., 2015), green tea waste (Siddiqui et al., 2016), olive pomace and olive leaf (Munekata et al., 2020; Difonzo et al., 2021), pomegranate peel (Chen et al., 2020) or aril, grape pomace or seeds (Silva et al., 2021), mango kernel (Mwaurah et al., 2020), myrtle berries seeds (Jabria et al., 2016), dates (Kchaou et al., 2016), walnut green husk (Jahanban-Esfahlan et al., 2019), almond skin (Bolling, 2017), tomato seeds (Taveira et al., 2010; Szabo et al., 2019), buckwheat hull extract (Cabarkapa et al., 2008), pomelo peel (Liu et al., 2017b; Tocmo et al., 2020).

Moreover, for a given plant, phenolics presence and/or concentration greatly varies from a plant tissue to another one: for instance, resveratrol concentration in vine (*Vitis vinifera*) was reported to vary from less than 1.5 mg/kg of fresh berries (where it is primarily located in skin) to 37 mg/kg in stems (Mikeš et al., 2008). As underlined by Piccolella et al. (2019) in their review, “isolating or detecting phenols and polyphenols necessarily requires a good knowledge of how, when and where these substances are bio-synthesized and stored.”

### Antimicrobial Activity of Individual Phenolics

Examples of antimicrobial phenolics belonging to the different structural groups of phenolics (phenolic acids, flavonoids, tannins, stilbenoids, quinones, and coumarins) as well as their minimal inhibitory concentrations (MICs) against various unwanted bacteria and their food sources are presented in Table 1. Examples of phenolic acids belonging to hydroxybenzoic acids and hydroxycinnamic acids are listed, as well as examples of flavonoids belonging to each of its different sub-classes according to their degree of oxidation: flavones, isoflavones, flavanones, flavonols, flavanols, and anthocyanins.

**Table 1:** Examples of antimicrobial plant phenolics belonging to phenolic acids, flavonoids, tannins, stilbenoids, quinones, and coumarins.

Phenolics group	Phenolics subgroup	Antimicrobial phenolic	Structure	Sensitive microorganisms and corresponding MIC^*^	References	Source
Phenolic acids	Hydroxybenzoic acids C_6_–C_1_	Gallic acid	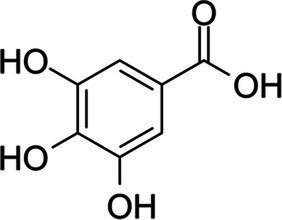	*Escherichia coli* 3.4 g·L^−1^ *Salmonella typhimurium* 3.4 g·L^−1^	Pacheco-Ordaz et al., 2017	Chestnut, clove		
				*E. coli* 1.5 g·L^−1^ *Pseudomonas aeruginosa* 0.5 g·L^−1^ *Staphylococcus aureus* 1.75 g·L^−1^ *Listeria monocytogenes* 2 g·L^−1^	Borges et al., 2013			
				*S. aureus* 0.21 g·L^−1^ *Bacillus cereus* 0.21 g·L^−1^ *E. coli* 0.21 g·L^−1^ *Salmonella* Infantis 0.21 g·L^−1^	Skroza et al., 2019			
		Vanillic acid	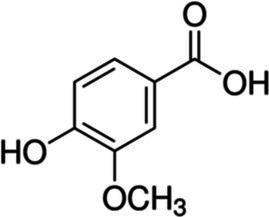	*E. coli*, 3.4 g·L^−1^ *S. typhimurium* 2.5 g·L^−1^	Pacheco-Ordaz et al., 2017	Açai oil, argan oil, wine, vinegar		
				*S. aureus* 0.42 g·L^−1^ *B. cereus* 0.1 g·L^−1^ *E. coli* 0.1 g·L^−1^ *S*. Infantis 0.21 g·L^−1^	Skroza et al., 2019			
		Proto-catechuic acid	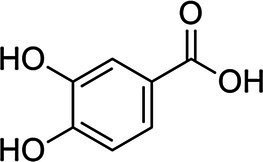	*E. coli*, 3.1 g·L^−1^	Pacheco-Ordaz et al., 2017	Bran and grain brown rice, olive oil, plums, gooseberries, white grapes, star anise, chicory, onion, almond		
				*S. aureus* 0.38 g·L^−1^ *B. cereus* 0.19 g·L^−1^ *E. coli* 0.38 g·L^−1^ *S*. Infantis 0.38 g·L^−1^	Skroza et al., 2019			
		Salicylic acid	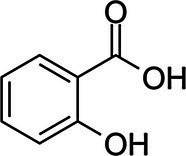	*S. aureus* 0.25–0.5 g·L^−1^ *Enterococcus faecalis* 0.5 g·L^−1^ *E. coli* 0.25–0.5 g·L^−1^ *P. aeruginosa* 0.5 g·L^−1^	Adamczak et al., 2020	Beer, coffee, tea, sweet potato, nuts, olive oil		
				*S. aureus* 0.35 g·L^−1^ *B. cereus* 0.17 g·L^−1^ *E. coli* 0.17 g·L^−1^ *S*. Infantis 0.17 g·L^−1^	Skroza et al., 2019			
		Syringic acid	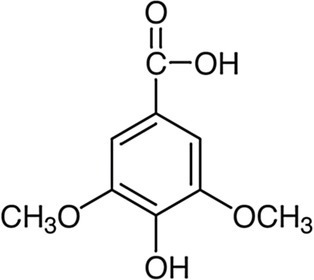	*S. typhimurium* 0.0625 g·L^−1^	Phadungkit and Luanratana, 2006	Olives, dates, pumpkin
				*S. aureus* 0.5 g·L^−1^ *B. cereus* 0.25 g·L^−1^ *E. coli* 0.25 g·L^−1^ *S*. Infantis 0.25 g·L^−1^	Skroza et al., 2019			
		Ellagic acid	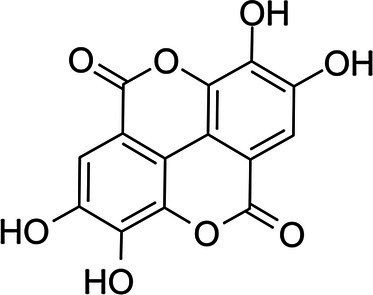	*S. aureus* 0.25 g·L^−1^ *Bacillus subtilis* 0.5 g·L^−1^ *Enterococcus. faecium* 0.25 g·L^−1^ *P. aeruginosa* 0.5 g·L^−1^ *E. coli* 0.25 g·L^−1^	An et al., 2021	Chestnut shells, berries, pomegranate, grape	
	Hydroxycinnamic acids C_6_–C_3_	Chlorogenic acid	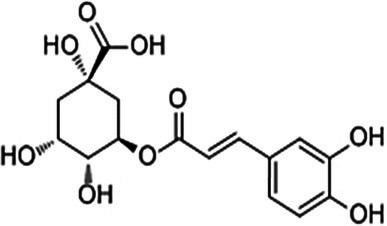	*E. coli* 0.5 g·L^−1^	Adamczak et al., 2020	Apple, artichoke, tea		
		Ferulic acid	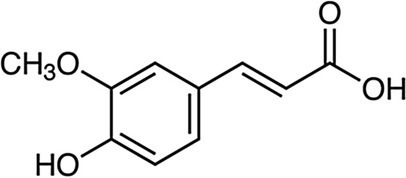	*E. coli*, 3.9 g·L^−1^ *S. typhimurium* 3.9 g·L^−1^	Pacheco-Ordaz et al., 2017	Wheat bran, rice bran, oat bran		
				*E. coli* 0.1 g·L^−1^ *P. aeruginosa* 0.1 g·L^−1^ *S. aureus* 1.1 g·L^−1^ *L. monocytogenes* 1.25 g·L^−1^	Borges et al., 2013			
				*S. aureus* 0.5 g·L^−1^ *B. cereus* 0.25 g·L^−1^ *E. coli* 0.5 g·L^−1^ *S*. Infantis 0.5 g·L^−1^	Skroza et al., 2019			
		Caffeic acid	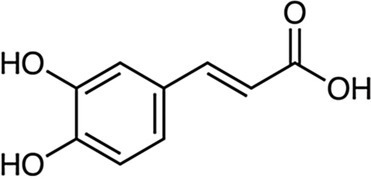	*E. coli* 0.25 g·L^−1^	Matejczyk et al., 2018	Sage, mint, Ceylon cinnamon, thyme		
				*S. aureus* 0.22 g·L^−1^ *B. cereus* 0.22 g·L^−1^ *E. coli* 0.11 g·L^−1^ *S*. Infantis 0.11 g·L^−1^	Skroza et al., 2019			
		Rosmarinic acid	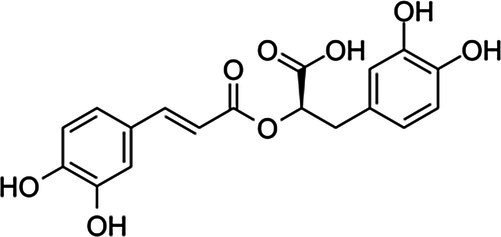	*E. faecalis* 1 g·L^−1^ *E. coli* 0.5 g·L^−1^ *P. aeruginosa* 0.5–1 g·L^−1^	Adamczak et al., 2020	Rosemary, lemon balm, oregano, sage, thyme		
				*S. aureus* 0.45 g·L^−1^ *B. cereus* 0.45 g·L^−1^ *E. coli* 0.45 g·L^−1^ *S*. Infantis 0.45 g·L^−1^	Skroza et al., 2019	
Flavonoids C_6_–C_3_–C_6_	Flavones	Luteolin	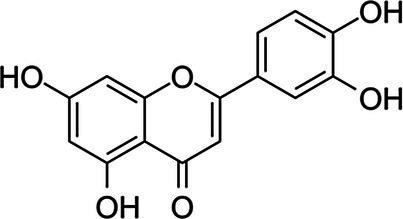	*S. aureus* 0.5 g·L^−1^ *E. faecalis* 1 g·L^−1^ *E. coli* 0.5 g·L^−1^ *P. aeruginosa* 0.5 g·L^−1^	Adamczak et al., 2020	Radicchio, peppers, lemon, pumpkin
				*S. aureus* 0.045 g·L^−1^ *B. cereus* 0.09 g·L^−1^ *E. coli* 0.09 g·L^−1^ *S*. Infantis 0.09 g·L^−1^	Skroza et al., 2019	
		Apigenin	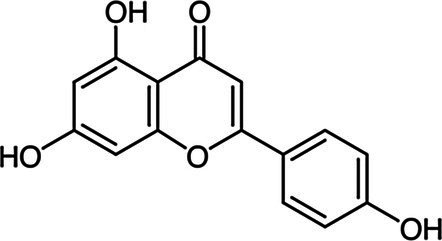	*S. aureus* 0.5–1 g·L^−1^ *E. faecalis* 1 g·L^−1^ *E. coli* 0.5 g·L^−1^ *P. aeruginosa* 0.5 g·L^−1^	Adamczak et al., 2020	Parsley, chamomile, celery, artichokes
		Flavone	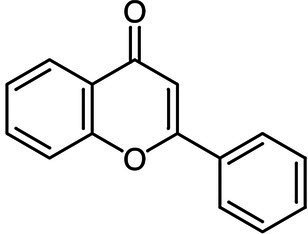	*E. faecalis* 0.5 g·L^−1^ *E. coli* 0.5 g·L^−1^ *P. aeruginosa* 0.5 g·L^−1^	Adamczak et al., 2020	Mandarin
		Vitexin, isovitexin, and vitexin 2"-o-rhamnoside	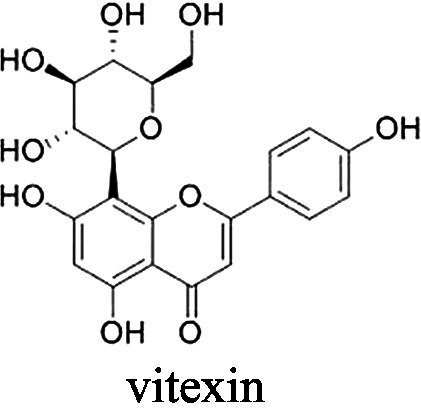 Vitexin	*E. faecalis* 1 g·L^−1^ *E. coli* 0.5 g·L^−1^ *P. aeruginosa* 0.5 g·L^−1^	Adamczak et al., 2020	Flaxseed, prairie turnip, mung bean
		Chrysin	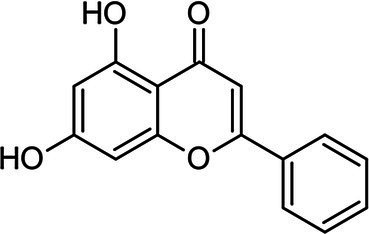	*S. aureus* 0.5 g·L^−1^ *E. faecalis* 1 g·L^−1^ *E. coli* 0.5 g·L^−1^ *P. aeruginosa* 0.5 g·L^−1^	Adamczak et al., 2020	Honey, propolis, carrots, chamomile
		Orientin and isoorientin	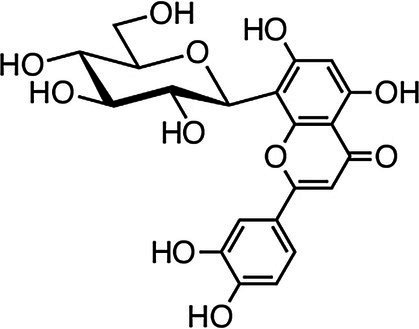	*S. aureus* 0.5 g·L^−1^ *E. faecalis* 1 g·L^−1^ *E. coli* 0.5 g·L^−1^ *P. aeruginosa* 0.5 g·L^−1^	Adamczak et al., 2020	Medicinal plants (e.g., bamboo leaves)
	Isoflavones	Phaseollidin	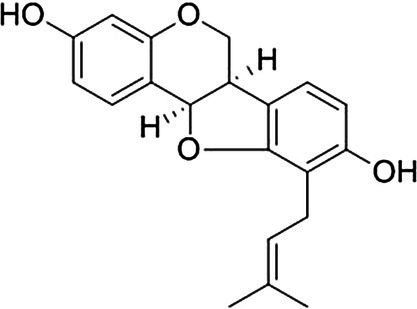	*B. cereus* 10 mg·L^−1^ *S. aureus* 10 mg·L^−1^ *E. coli* 20 mg·L^−1^ *P. aeruginosa* 20 mg·L^−1^	Sadgrove et al., 2020	*Erythrina* medicinal plants
	Flavanones	Naringin	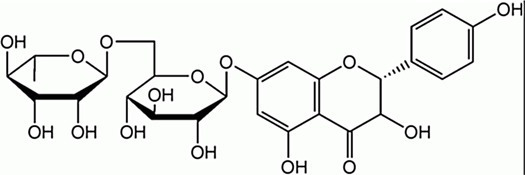	*E. faecalis* 1 g·L^−1^ *E. coli* 0.5 g·L^−1^ *P. aeruginosa* 0.5 g·L^−1^	Adamczak et al., 2020	Grapefruit, orange
	Flavonols	Kaempferol	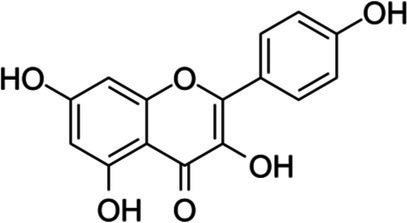	*E. coli* 0.5 g·L^−1^	Adamczak et al., 2020	Strawberry, spinach, broccoli
				*S. aureus* 0.09 g·L^−1^ *B. cereus* 0.09 g·L^−1^ *E. coli* 0.36 g·L^−1^ *S*. Infantis 0.36 g·L^−1^	Skroza et al., 2019	
				*E. faecalis* 0.1 g·L^−1^	del Valle et al., 2016	
		Quercetin	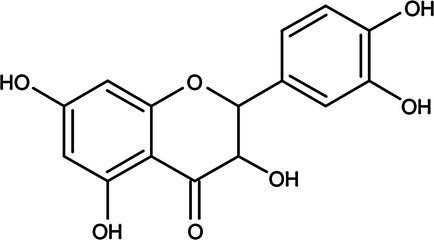	*E. coli* 0.5 g·L^−1^	Adamczak et al., 2020	Capers, red onion, grapes, berries, black and green tea
				*S. typhimurium* 15.6 mg·L^−1^	Phadungkit and Luanratana, 2006	
		Rutin	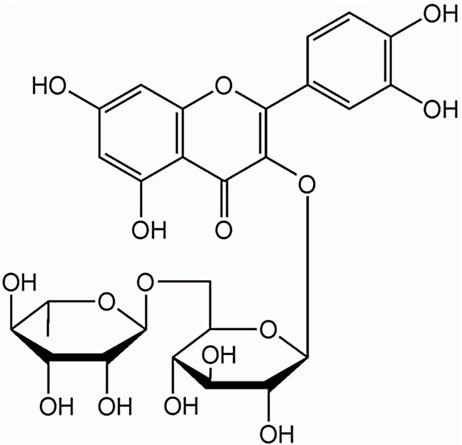	*S. aureus* 1 g·L^−1^ *E. faecalis* 1 g·L^−1^ *E. coli* 0.5 g·L^−1^ *P. aeruginosa* 0.5 g·L^−1^	Adamczak et al., 2020	Carob fiber, fennel leaves, parsley
	Flavanols	Catechin	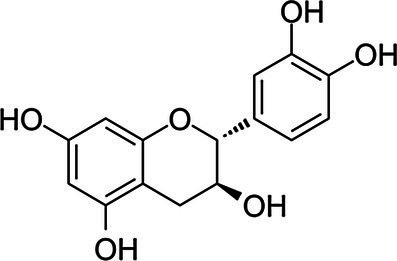	*S. aureus* 0.36 g·L^−1^ *B. cereus* 0.36 g·L^−1^ *E. coli* 0.18 g·L^−1^ *S*. Infantis 0.18 g·L^−1^	Skroza et al., 2019	Apple, apricot, cherry, peach berries, green tea
		Epicatechin	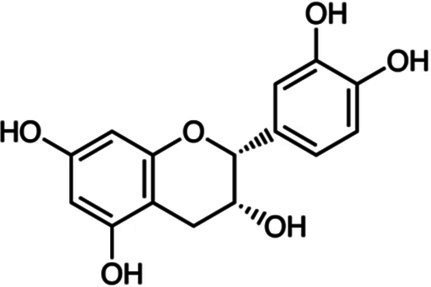	*S. aureus* 0.72 g·L^−1^ *B. cereus* 0.36 g·L^−1^ *E. coli* 0.72 g·L^−1^ *S*. Infantis 0.72 g·L^−1^	Skroza et al., 2019	Apple, blackberries
	Anthocyanins	Cranberry anthocyanins	n. d.	*S. aureus* 5 mg·L^−1^	Gong et al., 2021	Cranberry
Tannins		Tannic acid	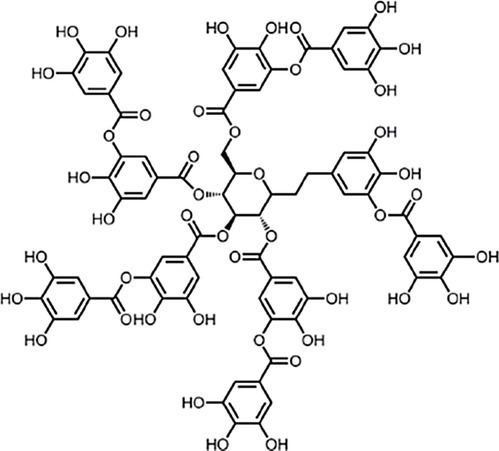	*S. aureus* 0.064 g·L^−1^	Kırmusaoğlu, 2019	Grape, green tea, persimmon
Stilbenoids		Resveratrol	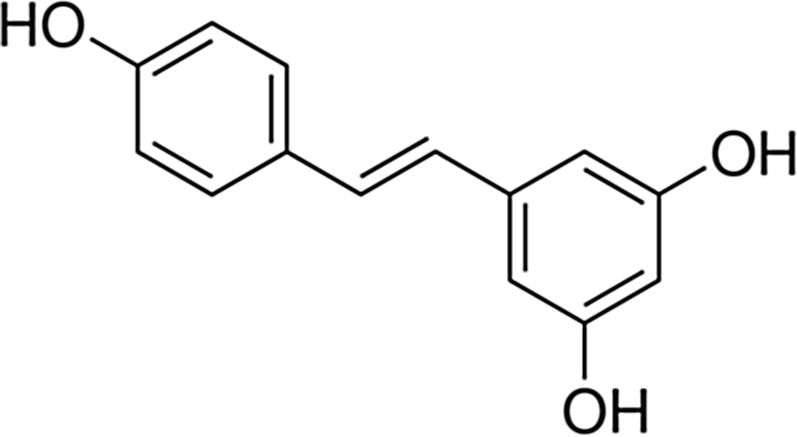	*S. aureus* 0.07 g·L^−1^ *B. cereus* 0.07 g·L^−1^ *E. coli* 0.14 g·L^−1^ *S*. Infantis 0.14 g·L^−1^	Skroza et al., 2019	Red grape, red wine, peanut butter, dark chocolate				
				*E. faecalis* 0.12 g·L^−1^	del Valle et al., 2016		
Quinones		2,6-dimethoxy-1,4-benzoquinone	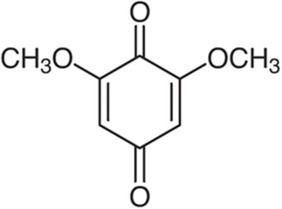	*S. typhimurium* 32 mg·L^−1^ *E. coli* 32 mg·L^−1^ *S. aureus* 8 mg·L^−1^ *B. cereus* 64 mg·L^−1^	Kim et al., 2010	Wheat germ
Coumarins		Scopoletin	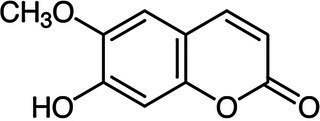	*S. typhimurium* 8 mg·L^−1^	Mfonku et al., 2021	Noni

*MIC, Minimal Inhibitory Concentration; n. d., not determined.

Significantly different MIC values of a given phenolic against the same bacterial species were sometimes reported by different authors. For instance, Pacheco-Ordaz et al. (2017) and Skroza et al. (2019) reported 3.4 g·L^−1^ and 0.21 g·L^−1^ MIC values of gallic acid against *Escherichia coli*, respectively. Such differences are likely due to the differences between the experimental procedures applied for MIC determination: in the absence of the use of a standard MIC determination procedure, differences regarding incubation conditions (initial number of culturable bacteria in the inoculum, temperature …) and bacterial strains can explain such differences. Indeed, MIC values were determined with *E. coli* O157:H7 ATCC 43890 and ŽMJ 129 (clinical isolate) strains, respectively. A 2.5 10^6^ cfu·ml^−1^ and a 10^5^–10^6^ cfu·ml^−1^ inoculum were added in Mueller Hinton broth, which was the incubated for 24 h at 37°C in both studies. Besides possible differences of sensitivity to gallic acid of both *E. coli* strains, a higher inoculum in the first case results thus in a significantly lower gallic acid to bacteria ratio, which might partly explain the highest MIC value reported by Pacheco-Ordaz et al. Another difference lies in the fact that in the first case MIC was detected by monitoring wells without turbidity, while in the second case absence of viable bacteria was detected following incubation with an indicator of bacterial respiratory activity (iodonitrotetrazolium chloride).

As underlined by Adamczak et al. (2020), differences can also result from the use of different solvents for the dissolution of pure phenolics although dimethylsulfoxide is generally used. However, as can be seen in Table 1, for most phenolics, MIC values determined by different authors against a given bacterial species are similar. The lowest MIC values reported in Table 1 are 5 mg·L^−1^ for cranberry anthocyanins against *Staphylococcus aureus* (Gong et al., 2021) and 8 mg·L^−1^ for scopoletin against *Salmonella enteritidis* serovar Typhimurium (Mfonku et al., 2021). Since anthocyanin contents of around 50 mg anthocyanins per 100 g of cranberries from different cultivars were reported (Narwojsz et al., 2019), cranberries are a promising source of such antimicrobial phenolics.

As pointed out by Adamczak et al. (2020), compared to the number of studies regarding the antimicrobial activity of phenolic-rich plant extracts, only a few authors investigated the antimicrobial activity of individual pure phenolics (Borges et al., 2013; Pacheco-Ordaz et al., 2017; Bouarab-Chibane et al., 2019; Skroza et al., 2019). Determination of the antimicrobial activity of pure phenolics commonly present in plant extracts opens the possibility to estimate their contribution to the activity of plant extracts, in which they are present: for instance, Phadungkit and Luanratana (2006), determined the MIC against *Salmonella* of the three phenolics identified in dried fruit extracts of *Ardisia elliptica* Thunb (syringin, quercetin and a methoxylated derivative of quercetin, isorhamnetin), a fruit used as food, as well as in Thai traditional medicine. Since the antimicrobial activity of plant extracts is generally due to different individual phenolics, antimicrobial activity assays of pure phenolics alone or in combination allows to check whether they have a synergistic activity. Gutiérrez-Fernández et al. (2013) observed thus a synergistic activity against *Enterococcus faecalis* of gallic acid and octyl gallate, while Mellegard et al. (2009) reported no synergistic activity against *S. aureus* of the four dominating phenolic compounds in the leaves of *Sphagnum papillosum*. Comparison of MIC against foodborne pathogenic bacteria of pure phenolics identified with their lower concentrations in the leaves of this plant led these authors to question their potency.

However, these studies with individual pure phenolics are generally performed with synthetic phenolics commercially available. This set of commercial molecules comprises a far lower number of phenolics than the tens of thousands of plant phenolics with different structures, that have been identified. For instance, plant phenolics with high molecular weight, such as tannins, are underrepresented. Moreover, most natural plant phenolics are glycosylated, while only a limited number of commercial phenolics are glycosylated (e.g., vitoxin, orientin, naringin, Table 1). Several authors reported that most aglycones of plant phenolics have a higher antimicrobial activity than their glycosylated forms. This is consistent with Guo et al. (2020) observation of an increase in antibacterial activity of mulberry leaves following their solid state fermentation by edible fungi, which was correlated with an increase in kempferol and quercetin aglycones at the expense of their glycosides.

Identification of antimicrobial phenolics in a plant is necessary to guide their extraction and get extracts with a standardized antimicrobial activity. The most active phenolics of plant extracts launched with a claim for their antimicrobial activity are thus 2,6-dimethoxy-1,4-benzoquinone in moso bamboo extract (Takeguard^™^, Takexlabo, Osaka, Japan), hydroxytyrosol in olive extract (Hidrox 10X Liquid Concentrate, CreAgri, Inc., Hayward, CA, United States). Nevertheless, it has been reported by many authors that extracts have generally a higher antimicrobial activity than a solution with only the most active phenolic at the same concentration. Serra et al. (2008) reported that a grape extract containing 20 mg·L^−1^ quercetin totally inhibited the growth of *Bacillus cereus* unlike a 20 mg·L^−1^ quercetin solution. Other constituents of grape extract exert thus an additive or synergistic effect with quercetin.

Antimicrobial activity assays of phenolics with different structures also allowed several authors to conduct Structure Activity Relationships (SAR) or Quantitative Structure Activity Relationships (QSAR) studies (Bouarab-Chibane et al., 2019). Since SAR mainly depends on the mechanisms of antimicrobial action on phenolics, the main conclusions of these studies are presented in the next section, after the diversity of mechanisms of actions of plant phenolics has been presented.

## Diversity of Antimicrobial Mechanism of Action of Plant Phenolics

### Overview of Known Antimicrobial Mechanisms of Action of Plant Phenolics

**Figure 1:**
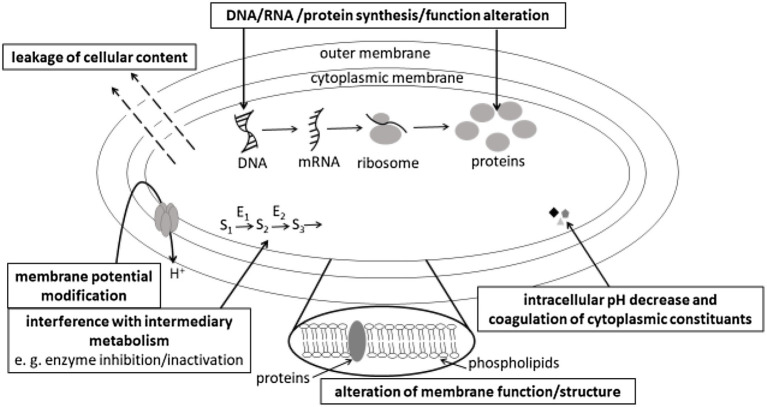
Main mechanisms of antibacterial action of plant phenolics.

Different mechanisms of action of plant phenolics active against bacteria, yeasts or fungi have been reported. The reader interested in the mechanisms of action against fungi and more specifically against mycotoxin production can refer to da Cruz Cabral et al. (2013) review. The main mechanisms of antibacterial action of plant phenolics described in this review are summarized in Figure 1. As it can be observed in Figure 1, the most commonly reported mechanisms of action of plant phenolics are at the membrane level. Many authors reported dose-dependent alterations from microbial membranes ranging from reversible membrane permeability perturbations to membrane disruption (inducing leakage of cellular content). However, as stated by Rempe et al. (2017) in their review, while monitoring influx of fluorescent dyes (e.g., propidium iodide can only enter cells with disrupted membrane), efflux of intracellular constituents or direct microscopical observation of microbial cells allow to check, whether their membrane was disrupted following treatment with plant phenolics, this does not give any indication regarding more specific mechanisms of action leading to membrane disruption: did the phenolic compound directly interact with membrane components or alter membrane stability by interfering with intracellular processes?

Nevertheless, flow cytofluorometric analysis of microbial cells stained with different fluorescent dyes allowing to monitor membrane permeability, membrane potential, intracellular pH or intracellular enzymes activity provides information on different functions affected by phenolics at the membrane level (Léonard et al., 2016).

A common proposed mechanism of action is based on the presence of -OH groups in phenolics structure, promoting interaction of phenolics by hydrogen-binding with the microbial cells envelope. Depending on their hydrophobicity, phenolics can accumulate at the surface of the cell envelope, penetrate or even cross their membrane and penetrate in the microbial cells cytoplasm, where they can interact with different cell constituents or alter intracellular pH. Gram-negative bacteria, which possess a hydrophilic cell wall, would be less sensitive to hydrophobic components of polyphenols than Gram-positive ones. Interestingly, Borges et al. (2013) compared the surface (hydrophobicity) and charge (zeta-potential) properties of two Gram-positive (*S. aureus* and *Listeria monocytogenes*) and two Gram-negative (*E. coli* and *Pseudomonas aeruginosa*) bacterial species following their treatment with either gallic or ferulic acids. Phenolic acids application increased the electron acceptor properties for Gram-positive bacteria and decreased these properties for Gram-negative ones. The surface charge of Gram-negative bacteria was less negative in the presence of the two phenolic acids, while that of Gram-negative bacteria was unchanged. These observations and the differences of susceptibility to phenolic acids of Gram-negative and Gram-positive bacteria support the assumption, that the differences in cell membrane structure and composition play a key role in the susceptibility to plant phenolics.

Undissociated forms of phenolic acids, prevailing at pH values below their pKa values, are uncharged and can thus cross the phospholipid bilayer of bacterial membranes and decrease intracellular pH (Wen et al., 2003; Pernin et al., 2019). Reported consequences of phenolics penetration in the cytoplasm of microorganisms encompass interruption of DNA, RNA, protein synthesis or functions, interference with intermediary metabolism [namely energy (ATP)-generating system], coagulation of cytoplasmic constituents resulting from its acidification. Mora-Pale et al. (2015) observed that resveratrol-trans-dihydromer inhibited DNA gyrase activity *in vitro*, and concluded from a transcriptomic analysis, that it downregulated ABC transporters, as well as genes involved in cell division and DNA binding proteins. Its bactericidal action against *B. cereus*, *L. monocytogenes*, *S. aureus*, and *E. coli* results both from membrane potential disruption and from DNA synthesis inhibition.

Phenolics can also interact with membrane proteins involved in different functions: Duggirala et al. (2014) reported that the coumarin, scopoletin, inhibited bacterial cell division protein, FtsZ. They proposed that the increased length of *Bacillus subtilis*, in the presence of this coumarin, was probably due to the absence of septum formation, resulting from the inhibition of the first step of bacterial cell division. Examples of antimicrobial plant phenolics or phenolic-rich plant extracts with different mechanisms of action are listed in Table 2.

**Table 2:** Examples of plant phenolics or phenolic-rich plant extracts with different antimicrobial mechanisms of action.

Plant phenolic or plant extract	Microorganism	Mechanisms of action	References
Nutgall (*Quercus infectoria*) extracts and their main constituents (namely gallic and tannic acids)	*S. aureus*	– no lysis but significant loss of tolerance to low osmotic pressure and high salt concentration following treatment with ethanol extract, one ethyl acetate fraction, gallic acid and tannic acid	Chusri and Voravuthikunchai, 2011
Ferulic acid or gallic acid (1 g·L^−1^, 30 min)	*L. monocytogenes* *E. coli* *P. aeruginosa*	– intracellular K^+^ efflux: membrane permeabilization	Borges et al., 2013
Berry phenolics	*Salmonella enterica* serovar Typhimurium *S. enterica* serovar Infantis	– can reduce outer membrane permeability in a similar manner to EDTA by releasing lipopolysaccharide (LPS) and chelating divalent cations or by intercalating into the outer membrane and replacing stabilizing cations	Nohynek et al., 2006
Ethanolic and water extracts of roselle (*Hibiscus sabdariffa*), rosemary (*Rosmarinus officinalis*), clove (*Syzygium aromaticum*), and thyme (*Thymus vulgaris*)	*S. aureus* *E. coli*	– decrease in internal pH and membrane hyperpolarization following treatment with extracts suggesting bacterial membrane damage	Gonelimali et al., 2018
*Satureja montana* and *Origanum majorana* decoctions (1.56 g·L^−1^)	*S. aureus*	– reversible alteration of membrane permeability following the first hours of exposure to *Satureja montana* and *Origanum majorana* decoctions	Gomes et al., 2020
Kombucha polyphenolic fraction	*Vibrio cholerae*	– fraction containing mainly catechin and isorhamnetin, as well as catechin and isorhamnetin permeabilizing the inner membrane of *Vibrio cholerae*	Bhattacharya et al., 2018
Chinese wild blueberries fraction with anthocyanins	*L. monocytogenes*, *S. aureus*, *S*. Enteritidis	– leakage of nucleic acids and proteins: membrane disruption	Zhou et al., 2020
Pinosylvin	Three *S. enterica* strains	– destabilization of the outer membrane of *Salmonella* cells partially abolished by MgCl_2_ addition indicating thus that part of its activity is related to the chelation of outer membrane stabilizing divalent cations, such as Mg^2+^	Plumed-Ferrer et al., 2013
3-p-trans-coumaroyl-2-hydroxyquinic acid from *Cedrus deodara*	*S. aureus*	– interaction with membrane lipid and protein, damage of cytoplasmic membrane with a significant membrane hyperpolarization, a loss of membrane integrity and severe morphological changes	Wu et al., 2016
*Backhousia citriodora* extract	*Saccharomyces cerevisiae*	– damage of the yeast cell membrane through penetration causing swelling and lysis leading to cell death	Alderees et al., 2018
p-hydroxybenzoic, protocatechuic, gallic, chlorogenic, vanillic, p-coumaric, and ferulic acids	*L. monocytogenes*	– decrease in extracellular pH (main mechanism of action for chlorogenic and gallic acids) – penetration or accumulation in the bacterial membrane of undissociated form (caffeic acid, p-hydroxybenzoic acid, protocatechuic acid, and vanillic acids) – dissociated form that is significantly antimicrobial (p-coumaric acid and ferulic acids)	Pernin et al., 2019
Olive leaf extract	*L. monocytogenes*	– loss of flagella and reduction of motility of *L. monocytogenes* cells following their treatment by sub-inhibitory concentrations of olive leaf extract	Liu et al., 2017a
Scopoletin and daphnetin (coumarins)	*Bacillus subtilis*	– increase in length of bacteria in the presence of the coumarins probably due to the lack of septum formation, hypothesis substantiated by screening for their ability to inhibit the bacterial cell division protein *Escherichia coli* FtsZ: – scopoletin inhibited the GTPase (GTP: guanosine triphosphate) activity of FtsZ in a noncompetitive manner – molecular docking studies of interactions of coumarins with the modeled FtsZ protein indicate that they bind to T7 loop, which is different from the GTP-binding site (active site) These data support the hypothesis of the role of coumarins in halting the first step of bacterial cell division process	Duggirala et al., 2014
Quercetin	*E. coli*	– DNA gyrase inhibition either by interaction of quercetin with DNA or with ATP binding site of gyrase	Plaper et al., 2003
Chlorogenic acid	*B. subtilis*	– induction of the intracellular metabolic imbalance of the tricarboxylic acid cycle and glycolysis, leading to metabolic disorder and death	Wu et al., 2020
Cranberry concentrate	*E. coli* O157:H7	– marked downregulation of *hdeA* (cell envelope protein), *slp* (outer membrane lipoprotein) and *cfa* (cell wall phospholipid synthesis) genes	Wu et al., 2009
Hydroxytyrosol	*Lactobacillus plantarum*	– upregulation of antioxidant response involving genes from the reactive oxygen species resistome of *Lb. plantarum*, genes coding for H_2_S-producing enzymes and genes involved in the response to thiol-specific oxidative stress – upregulation of a set of genes involved in cell wall biogenesis	Reverón et al., 2020
Gallic acid, protocatechuic acid and vanillic acid	*Salmonella enterica* serovar Typhimurium	– membrane permeabilization by the three phenolic acids – morphological defects at the polar ends of bacteria treated with gallic or protocatechuic acids – treatment by vanillic acid resulting in observation of mid-division cells suggesting a perturbation of the cell division process that allows for septum formation but prevents finalization – downregulation of all genes found in the Salmonella Pathogenicity Island 1 (SPI-1), which code for an assembly of proteins that aid in the attachment and subsequent invasion of host cells by gallic acid and downregulation of key regulatory genes by protocatechuic acid	Alvarado-Martinez et al., 2020
3-hydroxyphenylacetic acid (3-HPAA)	*Pseudomonas aeruginosa*	– proteomic analysis after 3-HPAA exposure of *P. aeruginosa* revealed changes in profile of proteins related to DNA replication and repair, RNA modifications, ribosomes and proteins, cell envelope, oxidative stress, as well as nutrient availability. 3-HPAA was thus classified as a multitarget antimicrobial agent	Ozdemir and Soyer, 2020

### Recent Progress and Future Prospects Regarding Antimicrobial Mechanism of Action of Plant Phenolics

Despite the diversity of mechanisms of action of antimicrobial plant phenolics listed in Table 2, this list is biased by the fact that most investigations were performed using target-directed bioassays [e.g., membrane permeabilization is evaluated by monitoring efflux of intracellular constituents, influx of dyes such as propidium iodide, or 1-N-phenylnaphthylamine (NPN) uptake, DNA gyrase B (gyrB) inhibition assays …], which preclude the discovery of other or even novel mechanisms of antimicrobial action (Rempe et al., 2017).

Nevertheless, multiplication in recent years of studies by -omics approaches opens the possibility to identify new mechanisms of action or to identify several mechanisms of action acting simultaneously (which is generally the case at least for plant extracts and even for some pure plant phenolics). Transcriptomics (Alvarado-Martinez et al., 2020; Linzner et al., 2020; Reverón et al., 2020), proteomics (Ozdemir and Soyer, 2020) and metabolomics (Wu et al., 2020) approaches recently contributed to the identification of targets of antimicrobial action of some plant phenolics or phenolic-rich plant extracts. Transcriptomic (Reverón et al., 2020) and metabolomic (Wu et al., 2020) analyses allow to identify which metabolic pathways are affected by plant phenolics.

Limitations of these -omics approaches are the identification and/or detection thresholds for different technologies, the availability of annotated information for microorganisms tested, and data interpretation difficulties. However, technological evolutions [e.g., more systematic use of high-resolution mass spectrometry (HRMS) for the identification of metabolites] help to solve these limitations. Another bias in past studies lies in the fact, that mechanisms of action were often investigated by treating target microorganisms with plant extracts or phenolics concentration above their MIC or even their Minimal Bactericidal Concentration (MBC). Such conditions often result in observing membrane disruption of target cells, which is the ultimate consequence of the action of some plant phenolics, but rarely the primary cause. It is thus also relevant to investigate the effect of sub-inhibitory concentrations of plant phenolics or extracts on different functions of target microorganisms. -Omics approaches also allow to investigate the effect of phenolics sub-inhibitory concentrations on the physiology of microorganisms.

Fungi are generally more resistant to plant phenolics than bacteria. However, despite their structural diversity, no clear general rule regarding the antimicrobial activity spectrum or the mechanisms of action of a class of plant phenolics could be established to date. Nevertheless, the interested reader can refer to several reviews or articles focused on the antimicrobial activity of each phenolic class: phenolic acids (Wen et al., 2003; Cueva et al., 2010; Borges et al., 2013; Pernin et al., 2019), flavonoids (Cushnie and Lamb, 2005), tannins (Scalbert, 1991), stilbenoids (Plumed-Ferrer et al., 2013), quinones (Linzner et al., 2020), and coumarins (Duggirala et al., 2014). Several authors also presented reviews on phenolic-rich plant extracts or plant phenolics acting on one (*L. monocytogenes*, Zamuz et al., 2021) or several (Ullah et al., 2020) foodborne pathogenic microorganisms.

### Structure-Antimicrobial Activity Relationships of Plant Phenolics

With the increasing number of antimicrobial phenolics with different structures identified, several authors investigated the relationships between phenolics structure and their *in vitro* antimicrobial activity (Taguri et al., 2006; Bouarab-Chibane et al., 2019). Taguri et al. (2006) compared the antibacterial activity of 22 polyphenols against 26 species of bacteria by determining their MICs in Mueller-Hinton broth. Bouarab-Chibane et al. (2019) investigated the effect of a 1 g·L^−1^ concentration of 35 polyphenols on the growth at 37°C for 24 h of three Gram-positive bacterial strains (*B. subtilis*, *S. aureus*, *L. monocytogenes*) and three Gram-negative ones (*E. coli*, *P. aeruginosa*, and *S*. Enteritidis). In both studies, the antibacterial activity was dependent on the species of bacteria and no significant difference of susceptibility to polyphenols was observed between Gram-positive and Gram-negative bacteria.

The fact that antibacterial activity of phenolics depends on the species of bacteria motivated some authors to perform QSAR studies focused on one bacterial species. For instance, Fang et al. (2016) built reliable QSAR models for the antibacterial activity against *E. coli* of 30 flavonoids. Moreover, molecular docking study of interaction with DNA gyrase B (gyrB) of *E. coli* allowed to establish that half of flavonoids active against *E. coli* inhibit gyrB by interacting with ATP binding site of this enzyme [the mechanism of antibacterial activity against *E. coli* of quercetin already proposed by Plaper et al. (2003)]. Pernin et al. (2018) proposed a QSAR model for the antibacterial activity against *L. monocytogenes* of 21 phenolics and found a good correlation with their octanol–water partition coefficient (log P_o/w_).

Taguri et al. (2006) observed that antibacterial activity of polyphenols having pyrogallol groups (i.e., with three adjacent hydroxyl groups) was higher than that of polyphenols with catechol and resorcinol rings (i.e., with two adjacent or non-adjacent hydroxyl groups, respectively). This is consistent with Phan et al. (2014) conclusion that “the higher number of hydrophilic side chains (hydroxyl, gallate, galloyl, glucoside), the more interactive the polyphenol was with the membrane” following observation of spatio-temporal real-time membrane dynamics in the presence of different polyphenols. Bouarab-Chibane et al. (2019) were not able to build reliable QSAR models predicting the effect of the set of 35 polyphenols they used on the growth of *L. monocytogenes* and *P. aeruginosa*, since they were systematically inhibited or not inhibited by polyphenols, respectively. However, reliable QSAR models, with a few independent physicochemical descriptors linked with lipophilicity and the electronic and charge properties of the polyphenols, were built for each of the four other bacterial strains. These physicochemical descriptors are consistent with the hypothesis that the main antibacterial mechanisms of action of polyphenols depend on their accumulation on the surface of bacteria, which is favored both by hydrogen binding and by their hydrophobicity.

For instance, the reduced susceptibility to polyphenols like epigallocatechin gallate of many lactic acid bacteria compared to other Gram-positive bacteria was proposed to result from exopolysaccharides production, which reduces surface hydrophobicity of lactic acid bacteria (Nakayama et al., 2015). Therefore, Bouarab-Chibane et al. (2019) investigated the surface properties of the six species of test bacteria they used by Microbial Adhesion To Solvents (MATS), following the method described by Bellon-Fontaine et al. (1996). Interestingly, they noted that bacteria, which had a similar affinity for the four solvents they used, had similar QSAR models, while the bacteria which had different affinity for these solvents also had different QSAR models. Since only four bacterial strains had reliable QSAR models, they suggested evaluating, whether this trend would also be observed with a larger number of bacterial strains. Moreover, characterization of the surface properties of bacteria should also integrate other parameters such as their zeta-potential.

Besides investigating the structure-antimicrobial activity relationships of phenolics naturally present in plants, several authors performed SAR or QSAR studies with series of epicatechin gallate groups, from which gallate group was substituted with 3-O-acyl chains of varying lengths (C_4_–C_18_; Stapleton et al., 2004), or with series of esters of phenolic acids esterified with various alkyl or aryl substituents (Andrade et al., 2015; Shi et al., 2018; Araujo et al., 2019). This allowed them both to get further insight in the physicochemical characteristics of active antimicrobial phenolics and to design new antimicrobial phenolics.

## Antimicrobial Activity of Plant Phenolics in Foods: Interest, Limits and Future Prospects

### Examples of Antimicrobial Action of Plant Phenolics in Different Food Matrices

The potential of several phenolic-rich plant extracts or pure plant phenolics to inhibit the growth of unwanted microorganisms in some food matrices has been demonstrated in many studies. Some recent examples are listed in Table 3. As illustrated by these examples, many studies aimed at improving the preservation of meat and meat products, likely since these foods both have a high added-value and are highly perishable. Application of phenolic-rich plant extracts to meat and meat products preservation has been recently reviewed (Efenberger-Szmechtyk et al., 2021). Moreover, addition of herbs and spices in meat and meat products is a common practice to improve their sensory properties. As stated in Table 3 (e.g., Ranucci et al., 2019), plant phenolics and phenolic-rich plant extracts addition in meat and meat products has frequently been reported to delay oxidation reactions altering organoleptic properties of such foods (e.g., color alteration due to myoglobin oxidation into metmyoglobin or taste or aroma alteration due to fat oxidation).

**Table 3:** Examples of phenolic-rich plant extracts or plant phenolics effectively inhibiting the growth of unwanted microorganisms in food matrices.

Plant phenolic or plant extract	Food supplemented with plant phenolic or plant extract	Effects on food quality	References
Clove extract	Raw porcine meat supplemented with clove extract [concentration of clove extract expressed in g of powdered clove used for the extraction per 100 g of meat was 0.5% (w/w)]	– clove extract decreased the growth of total viable count, *Pseudomonas*, *Enterobacteriaceae* during storage at 4°C but did not increase shelf life of pork meat	Muzolf-Panek et al., 2019
*Rumex tingitanus* extract with a high luteolin content	Raw bovine minced meat supplemented with 10, 20 and 30 mg of *R. tingitanus* extract/g of meat	– bactericidal effect following inoculation of *L. monocytogenes* (2.10^2^ cfu/g of meat) and inhibition of growth of mesophilic and psychrotrophic bacteria from meat during storage at 4°C for 30 days	Mhalla et al., 2017
White cabbage (*Brassica oleracea* var. *capitate* f. *alba*) extract	Raw bovine meat	– white cabbage extracts addition to meat [at either a 0.5% or a 1% (w/w) concentration] decreased total viable counts, psychrotrophic bacteria, yeasts and molds over 16 days storage at 4°C	Rubab et al., 2020
*Hibiscus sabdariffa* L. ethanolic extract	Raw bovine meat sprayed with 0.25 g·L^−1^ to 1.25 g·L^−1^ *H. sabdariffa* ethanolic extract	– decreased growth of mesophilic and psychrophilic bacteria over 15 days storage at 4°C	Márquez-Rodríguez et al., 2020
Green tea, stinging nettle and olive leaves extracts	Frankfurter sausage	– plant extracts (0.5 g/kg of sausage) reduced the count of total viable bacteria, mold and yeast by at least 2 log cfu/g over 45 days storage at 4°C	Alirezalu et al., 2017
Pomegranate (*Punica granatum*) and *Citrus* spp. extract mix	Sausage made from pork meat, emmer wheat (*Triticum dicoccum* Schübler), almond (*Prunus dulcis* Mill.), and hazelnut (*Corylus avellana* L.)	– addition of 0.5% or 1% (w/w) mix in the sausages delayed the pH drop, oxidation, total viable count, lactic acid bacteria and psychrotrophic microbial counts and extended the estimated shelf life of vacuum_packaged cooked sausages stored at 4°C from 44 to 50 or 60 days, respectively	Ranucci et al., 2019
p-coumaric acid, caffeic acid, and rutin	Chicken soup	– for concentrations above 0.23% (w/v), each phenolic totally inhibited the growth of *S. aureus* inoculated in chicken soup at 4°C within 3 days	Stojkovic et al., 2013
Cranberry extract or oregano extract	Seafood products (cod fish fillets and shrimps)	– inhibition of growth of *Vibrio parahaemolyticus* inoculated on fish fillets or shrimps (10^3^ cfu/g of seafood) during storage at 4°C for 8 days	Lin et al., 2005
*Perilla frutescens* leaf ethanolic extract	Surimi fish balls	– addition of 0.03% (w/w) extract into surimi fish balls decreased lipid and protein oxidation, formation of total volatile basic nitrogen and growth of *E. coli* during storage at 4°C – improved sensory properties	Zhao et al., 2019
*Quercus infectoria* ethanolic extract	Pasteurized bovine milk	– addition of extract to pasteurized milk resulted in lower total bacterial and yeast-mold counts and higher pH compared to control milk	Başyiğit et al., 2020
Olive mill wastewater extract	Fior di latte cheese	– addition of 250 mg/L or 500 mg/L of olive mill wastewater polyphenols in batches of Fior di latte cheese retarded *Pseudomonas fluorescens* and *Enterobacteriaceae* growth resulting in a 2 days and 4 days shelf life extension, respectively	Roila et al., 2019
Broccoli by-products extract	Fresh-filled pasta	– addition of 10 to 20% (v/w) of phenolic-rich broccoli extract in ricotta and spinach-based filling of fresh pasta resulted in a decrease in mesophilic bacteria growth during subsequent storage at 4°C thereby contributing to an extension of shelf life from 6 to 24 days	Angiolillo et al., 2019
Olive mill wastewater extract	Bread	Olive polyphenols emulsion (200 mg/kg) extended the shelf life of bread from 10 to 15 days	Galanakis et al., 2018
Olive leaf extract	Ready-to-use olive-based pâté	– addition of 0.5 or 1 g olive leaf extract.kg^−1^ of pâté resulted in a significant loss of *ca*. 0.5–1 logarithmic cycles of main microbial groups in samples added with extract, especially with 1.0 g·kg^−1^ during storage at 4°C under argon-based atmosphere for 120 days	Difonzo et al., 2019
Mulberry leaf extract	Fresh-cut cantaloupe	– spraying of fresh-cut cantaloupe with 5 g·L^−1^ mulberry leaf polyphenols resulted in a significant decrease in bacterial counts over 4 days of subsequent storage at 25°C compared to a control sprayed with sterile water	Yu and Shi, 2021

However, addition of plant phenolics or phenolic-rich plant extracts to meat and meat products and other food matrices has frequently more positive effects on retardation of oxidation phenomena than on retardation of the growth of unwanted microorganisms. For instance, Bouarab-Chibane et al. (2017) added 1% (w/w) of antimicrobial phenolic-rich plant extracts to raw bovine meat patties, enumerated bacteria and monitored different quality attributes of meat during 12 days storage at 4°C in a high-oxygen modified atmosphere. While pomegranate peel, green tea leaves, grape seed extracts, and Gaillac red wine powder reduced the increase in ThioBarbituric Acid Reactive Substances (TBARS are namely aldehydes generated following the decomposition of lipid peroxidation products) in bovine patties over 12 days by more than 93%, these extracts had no significant effect on total viable and psychrotrophic bacterial counts, despite their *in vitro* bactericidal activity against several Gram-positive and Gram-negative bacteria at a 0.1% (w/w) concentration. This apparent discrepancy can be due to the absence of susceptibility to these extracts of total viable and psychrotrophic bacterial strains, resulting from the diversity of strains in the meat microbial ecosystem. However, it could also be due to the interactions of antimicrobial plant molecules with proteins and/or fat which are the main constituents of bovine meat: these interactions could limit the quantity of “free” phenolics not interacting with these food constituents.

Nevertheless, despite their generally lower *in vitro* antimicrobial activity than in microbiological media, several authors reported plant phenolics or phenolic-rich plant extracts effectively inhibiting the growth of foodborne pathogenic micrororganisms [e.g., *L. monocytogenes* (Mhalla et al., 2017), *S. aureus* (Stojkovic et al., 2013)] or of food-spoiling microorganisms [e.g., *Pseudomonas*, *Enterobacteriaceae* (Muzolf-Panek et al., 2019)] in foods such as meat products. Mhalla et al. (2017) reported that incorporation of a luteolin-rich *Rumex tingitanus* extract in raw bovine minced meat retarded the growth at 4°C of mesophilic and psyschrophilic bacteria and addition of a pomegranate and *Citrus* spp. extracts mix in the formulation of pork sausages resulted in a significant extension of their shelf life (Ranucci et al., 2019). The potential of direct addition of plant extracts in other perishable animal origin foods containing proteins and fat than meat was investigated by other authors: addition of *Perilla frutescens* leaf extract into surimi fish balls decreased growth of *E. coli* during storage at 4°C (Zhao et al., 2019) and addition of olive mill wastewater polyphenols in batches of Fior di latte cheese retarded *Pseudomonas fluorescens* and *Enterobacteriaceae* growth resulting in significant shelf life extension (Roila et al., 2019). Addition of plant extracts at concentrations exceeding 1% (w/w) are generally required to effectively inhibit the growth of unwanted microorganisms in foods. It might even be more promising to add phenolic-rich plant extracts in foods containing lower amounts of food constituents interacting with proteins and fat, such as fruits, vegetables or bread (Galanakis et al., 2018).

Interestingly, non-minced meat pieces or fish fillets can be treated by spraying plant extracts or phenolics solutions on their surface, that is precisely where microbial contaminations and growth occur during storage. For instance, Márquez-Rodríguez et al. (2020) reported that spraying raw bovine meat pieces with a ~ 1 g·L^−1^ *Hibiscus sabdariffa* extract solution decreased the growth of mesophilic and psychrophilic bacteria over 15 days subsequent storage at 4°C. Similarly, Lin et al. (2005) voluntarily inoculated seafood with *Vibrio parahaemolyticus* and observed that spraying cranberry and oregano extracts solutions on their surface inhibited its growth for 8 days storage at 4°C. This strategy is also promising for fresh-cut fruits preservation as recently illustrated by Yu and Shi (2021) who sprayed fresh-cut cantaloupe with 5 g·L^−1^ mulberry leaf polyphenols to inhibit bacterial growth on their surface.

### Limits to the Application of Plant Phenolics for Food Preservation

#### Effect of Interaction With Food Constituents on Plant Phenolics Antimicrobial Activity

Besides interactions of phenolics with food constituents such as proteins or fat and resulting decrease in their “free” amount available to act on target microorganisms, unwanted microorganisms could also be protected by a “layer” of food constituents limiting the direct contact of antimicrobial phenolics with their cell envelope, the most commonly described mechanism of action of antimicrobial phenolics: da Cruz Cabral et al. (2013) reported that “lipids in food could form a coating around the microorganisms, protecting them from antimicrobial agents.” Boziaris et al. (2011) also reported the absence of effect on viable populations of spoilage or pathogenic bacteria were found between fish flesh or fish roe (tarama salads) treated or not with 10% (v/w) of *Filipendula ulmaria* liquid extract. For instance, *L. monocytogenes* Scott A was not affected by 10% (v/w) *F. ulmaria* extract over 12 days storage at 5°C of seafoods inoculated by this strain, while it was inhibited by the same concentration of this plant extract on solid microbiological medium also incubated at 5°C to better mimic food preservation conditions. They proposed that the loss of susceptibility of *L. monocytogenes* is due to interactions of *F. ulmaria* extract active phenolics (e.g., caffeic, p-coumaric and vanillic acids, myricetin …) with proteins and fat (the main constituents of such foods), at the expense of their interactions with unwanted microbial cells.

Indeed most studies regarding the antimicrobial activity of plant phenolics have been performed *in vitro* in microbiological media with a less complex composition and microstructure than food matrices. Miceli et al. (2014) compared the *in vitro* activity of borage (*Borago officinalis*) and Indian mustard (*Brassica juncea*) aqueous extracts against several foodborne pathogenic bacterial strains *in vitro* in Brain Heart Infusion broth and in meat, fish and vegetable broths (considered as food models). They observed that a 10-fold higher concentration than the *in vitro* bactericidal concentration was necessary to get a bacteriostatic effect in food models for both extracts. They suggested that this difference could be due to binding of the active compounds of extracts to food components.

Carbohydrates, lipids and proteins are the main components of food, and interactions between polyphenols and these components have been reported by many authors and reviewed by Jakobek (2015). Most studies regarding interactions between polyphenols and these components aimed at investigating their effect on their bioavailability following their ingestion. Interactions between phenolics and proteins are the most studied for this reason, and because interactions between compounds such as tannic and gallic acids and salivary proteins are involved in their astringency. Xu et al. (2018) compared thus the MICs against different microorganisms of epigallocatechin gallate and grape seed extract in a protein-free chemically defined medium and in the same medium supplemented with up to 0.4 g·L^−1^ of bovine serum albumin. A 64-fold increase in MIC of epigallocatechin gallate against a *Streptococcus mutans* strain was observed and a similar trend was observed with grapeseed extract, as well as against other microbial species. Such an increase in MIC was not observed for non-phenolic antimicrobial compounds, which were tested in parallel.

Besides protein content, which is far higher in most perishable foods than in microbiological media, another important difference between *in situ* antimicrobial activity assays and *in vitro* ones lies in the physiological state of microorganisms under refrigeration conditions and at optimal temperatures for microbial growth (i.e., between room temperature and 37°C), respectively. Klancnik et al. (2011) compared the activity in buffered peptone water supplemented with 50% (w/v) of meat, vegetable or dairy products to estimate the effect of the components of these foods on the activity against *L. monocytogenes* and *E. coli* of rosemary phenolic extracts. They also observed an increase in MIC against these two bacterial species for every food type added in buffered peptone water.

Therefore, Bouarab-Chibane et al. (2018b) compared the antibacterial activity of five bacteriostatic or bactericidal phenolics active against a *S. aureus* strain for 24 h at 37°C in Mueller-Hinton broth, with their activity in the same medium supplemented with up to 20% (w/w) bovine meat proteins (the protein content of bovine meat) for up to 8 days at 6°C (to mimic refrigeration conditions). While resveratrol and chrysin always lost their bacteriostatic activity in the presence of bovine meat proteins, gallocyanin kept its bactericidal activity at 37°C up to a 5% (w/w) protein content in the medium, but not at 15°C or 6°C, unlike naphthazarin, which was bactericidal at 6°C and 15°C, unlike at 37°C in the presence of bovine meat proteins. Finally, isobutyl-4-hydroxybenzoate kept its bactericidal activity under all the conditions investigated. The partition coefficient at 6°C of each phenolic between a 20% (w/w) bovine meat extract suspension and the same suspension without proteins was determined. Interestingly, the antibacterial activity reduction of phenolics in the presence of bovine meat proteins was correlated with their affinity for bovine meat proteins. Ansari et al. (2015) also reported that antimicrobial activity loss in the presence of casein or gelatin was correlated with their affinity for these proline-rich proteins.

It is thus advisable to perform at an early stage an *in vitro* screening of antimicrobial activity of phenolic-rich plant extracts in media containing proteins, if an application to foods containing proteins is foreseen (Bouarab-Chibane et al., 2018c), and preferably with the same proteins as in food. Screening at refrigeration temperatures is also preferable, if application to preservation of perishable foods is foreseen. Antimicrobial activity of plant extracts can be greatly influenced by temperature, as underlined by Hayrapetyan et al. (2012). They reported a higher inhibition of *L. monocytogenes* in meat pâté by pomegranate peel extract at 4°C than at 7°C, or 12°C. More systematic studies on the effect of food ingredients on the antimicrobial activity of phenolic-rich plant extracts or plant phenolics under conditions closer to food preservation conditions than classical *in vitro* antimicrobial activity screening assays should contribute to anticipate their *in situ* activity in real foods. While studies regarding the effect of proteins on the antimicrobial activity of plant phenolics exist, studies regarding the effects of carbohydrates or lipids on their activity are scarce and will also be necessary, since these compounds also interact with polyphenols. In the absence of such data, the efficiency of a given plant extract or phenolic to preserve a defined food can hardly be extrapolated to other foods differing in their composition and/or microstructure.

#### Effect of Plant Phenolics Addition on Foods Organoleptic Properties

Another important limit of many plant extracts is their effect on organoleptic properties of foods: the taste (e.g., astringency of some tannins, bitterness of green tea extract), odor and/or color (e.g., green color of green tea extract) of many plant extracts can alter the organoleptic quality of some foods (Bouarab-Chibane et al., 2017). However, in some cases, plant extracts added in concentrations effectively inhibiting the growth of unwanted microorganisms can also improve the color or the taste of some foods. Swer et al. (2019) added up to 0.2% (w/v) of anthocyanins extracted from Sohiong (*Prunus nepalensis* L.) in yogurts. Increase in anthocyanins content improved overall acceptability of yogurts by panelists. It is also possible to use plant extracts from which coloring substances have been removed. For instance, Nirmal and Benjakul (2011) added green tea ethanolic extracts without chlorophyll to Pacific white shrimps. One advantage of antimicrobial pure phenolics over plant extracts might also result from their absence of effect on taste of foods at a dose effectively inhibiting unwanted microorganisms, as underlined by Stojkovic et al. (2013) for chicken soup and porcine meat preserved by incorporation of 1.87 g·L^−1^ or spraying of 1.87 mg.10 cm^−2^ of phenolics (p-coumaric acid, caffeic acid, or rutin). However, as stated by Albuquerque et al. (2021), only the use of following pure phenolics is authorized in foods: anthocyanins [E 163, authorized as colorant by European Food Safety Authority (EFSA in the European Union) or Food and Drug Administration (FDA in the United States)] and ferulic acid (as antioxidant in Japan).

### Prospects for a Broader Use of Plant Phenolics for Food Preservation

Another potential advantage of the addition of some plant extracts or phenolics lies in their health-promoting properties. Since health-promoting properties of edible plant extracts or phenolics are not in the scope of the present review, the interested reader can refer to a recent review on this subject (Samtiya et al., 2021). As stated above, no plant extract will have the capacity to replace synthetic food preservatives, such as potassium sorbate as antifungal agent, in all their applications for the same cost. This is also namely due to the narrower antimicrobial activity spectrum of plant extracts compared to most food preservatives. A solution to have a broader antimicrobial activity spectrum is to use mixtures of plant extracts with different antimicrobial activity spectra. This is likely one of the reasons for the better preservation of raw bovine meat by mixtures of clove, cinnamon and oregano extracts rather than each of these extracts alone, or binary combinations of these extracts reported by Radha Krishnan et al. (2014). The narrow spectrum of antimicrobial activity of phenolic-rich plant extracts can also be positively exploited in some cases. For instance, the fact that lactic acid bacteria are generally less susceptible to the antibacterial plant phenolics compared to most undesirable bacteria (Pacheco-Ordaz et al., 2017; Chan et al., 2018) opens the possibility to add phenolic-rich plant extracts in foods fermented by lactic acid bacteria at sub-inhibitory concentrations of lactic acid bacteria, while effectively inhibiting the growth of unwanted microorganisms.

Another promising strategy to expand the application of phenolic-rich plant extracts is in line with hurdle technology principles application: appropriate combinations of phenolic-rich plant extracts addition, with for instance the addition of organic acids (Apostolidis et al., 2008), inoculation of food with bioprotective microorganisms (Sireswar et al., 2017), modified atmosphere or vacuum (Ranucci et al., 2019) packaging, high hydrostatic pressure (Hygreeva and Pandey, 2016) or UV-A treatment (Cossu et al., 2018) of foods have for instance been used to get a synergistic antimicrobial activity. Sireswar et al. (2017) inoculated seabuckthorn juice supplemented with malt extract with probiotic lactic acid bacteria strains. They demonstrated that growth of probiotic strains was possible thanks to malt extract addition and despite antibacterial phenolics presence in seabuckthorn juice. Moreover, they demonstrated the capacity of this food to rapidly eliminate different enteropathogenic bacteria: they proposed that the rapid elimination of these pathogens results from the synergistic action of metabolites such as lactic acid produced by probiotic bacteria and antimicrobial phenolics from seabuckthorn juice, such as isorhamnetin, myricetin, kaempferol, and quercetin.

## Diversity of Mechanisms of Action Against Biofilms of Plant Phenolics

### Overview of Known Mechanisms of Action of Plant Phenolics Against Biofilms

Biofilms confer favorable growth environments to pathogens and resistance to antimicrobials and disinfectants (Oulahal et al., 2008). Biofilms formed by undesirable bacteria are a major concern for food microbial safety, due to production of virulence factors, persistence of bacterial pathogens, cross contamination causing many risks for consumers. Mishra et al. (2020) recently reviewed strategies to control biofilm-forming pathogens based on the exploitation of natural anti-biofilm agents, including phytochemicals such as plant antimicrobial phenolics. Guzzo et al. (2020) more specifically reviewed the activity against *P. aeruginosa* and *S. aureus* biofilms of plant-derived natural products and Slobodníková et al. (2016) reviewed antibiofilm activity of plant polyphenols. However, these three reviews were focused on their clinical application in the medical and healthcare sectors and not in the food sector. The reader interested in plant phenolics potential against biofilm-forming food-contaminating microorganisms can refer to the review by Takó et al. (2020).

Briefly, increased tolerance to antimicrobial compounds of microorganisms present in biofilms results from different phenomena: microbial cells in biofilms are embedded in self-secreted extracellular polymeric substances (Sivaranjani et al., 2016), that attach these cells. These extracellular polymeric substances are namely polysaccharides, proteins and extracellular DNA. They protect cells from desiccation, pH variations but also from antimicrobial molecules including plant phenolics. They act as a protective layer of the surface of cells, as viscosifying agents slowing down the penetration and the diffusion of antimicrobial molecules in biofilms structure, thereby favoring adaptation of microbial cells, which have more time to become tolerant. Besides this “physical tolerance” favored by extracellular polymeric substances, “physiological tolerance” to antimicrobial molecules of microbial cells embedded in the deepest layers of biofilms has also been reported (Mishra et al., 2020). Indeed, important decreasing gradients of nutrients or oxygen result in the downregulation of the metabolic activity of microbial cells located inside the structure of biofilms. These reversible adaptative stress responses make these cells more tolerant to antimicrobial molecules. That is why these slow-dividing cells are called persister cells. Progresses in -omics (genomics, transcriptomics and metabolomics) allow identification of molecular pathways leading to biofilm formation and will contribute to get information for the rational design of efficient strategies to control biofilm formation.

Knowing the different stages of formation and development of biofilms also guides the design of strategies to prevent biofilms formation or to favor their eradication. Indeed, biofilm formation (i) starts with the attachment of microbial cells to a surface, is followed by (ii) biofilm structure development, (iii) its maturation and finally, (iv) its dispersion. Since attachment involves cytoskeletal elements, such as flagella and lipopolysaccharides, molecules inhibiting their formation are particularly promising to prevent their formation. Many authors recently reported the inhibition of biofilm formation by phenolic-rich plant extracts/plant phenolics by different mechanisms of action. A survey of such studies illustrating the diversity of these mechanisms of action is provided in Table 4.

**Table 4:** Examples of plant phenolics or phenolic-rich plant extracts acting against biofilms by different mechanisms of action.

Plant phenolic or plant extract	Microorganism	Mechanisms of action	References
*Moringa oleifera* extract	*P. aeruginosa*	– growth inhibition at a 0.05 mg·ml^−1^ concentration, while biofilm formation was reduced by 88% after 24 h	Onsare and Arora, 2015
3-p-trans-coumaroyl-2-hydroxyquinic acid from pine needles of *Cedrus deodara*	*S. aureus*	– inhibits the biofilm formation of *S. aureus* by affecting the initial attachment phase of biofilm development	Wu et al., 2016, 2019
Olive mill waste (olive vegetation water) extract	*Escherichia coli* K12	– olive vegetation water extract sub-inhibitory concentrations decreased biofilm formation, swarming and swimming motility – repression of genes for flagellar synthesis and of other genes linked to biofilm formation was observed	Carraro et al., 2014
Ethyl acetate fraction of *Adenanthera pavonina* ethanolic extract	*P. aeruginosa* PAO1	– 0.1 mg·ml^−1^ ethyl acetate fraction of *A. pavonina* ethanolic extract inhibits swarming motility of *P. aeruginosa* PAO1 – 0.5 mg·ml^−1^ ethyl acetate fraction of *A. pavonina* ethanolic extract inhibited pyocyanin production by *P. aeruginosa* PAO1 – viability of *P. aeruginosa* PAO1 was not affected at the tested concentrations of ethyl acetate fraction of *A. pavonina* ethanolic extract as observed by cell count	Vasavi et al., 2015
Dichloromethane fraction of ethanolic extract of *Camellia nitidissima* Chi flowers	*P. aeruginosa* PAO1	– fraction of *C. nitidissima* Chi flowers extract inhibits pyocyanin production by *P. aeruginosa* without affecting its growth – fraction of *C. nitidissima* Chi flowers extract exerts a concentration-dependent inhibitory activity of swarming and swimming motility of *P. aeruginosa*	Yang et al., 2018
*Allium sativum* extracts: raw garlic extract, heated garlic extract and toluene extract	*P. aeruginosa* PAO1	– inhibits biofilm formation and exerts anti-quorum sensing activities against *P. aeruginosa* PAO1 at sub-inhibitory concentrations (i.e. below MIC)	Vadekeetil et al., 2015
*Rosa rugosa* tea polyphenolic extract	*P. aeruginosa* PAO1 *Escherichia coli* K12	– inhibits swarming motility and biofilm formation of both strains in a concentration-dependent manner – inhibited quorum-sensing controlled violacein production in *Chromobacterium violaceum* (a quorum sensing - controlled phenotype) This extract is proposed to be a quorum-sensing inhibitor and/or anti-biofilm agent	Zhang et al., 2014
*Heracleum orphanidis* methanolic and ethanolic extracts	*P. aeruginosa* PAO1	– inhibit biofilm formation at sub-inhibitory concentrations – reduce the twitching and flagella mobility – reduces pyocyanin production Taken together, these observations suggest an inhibition of *P. aeruginosa* PAO1quorum sensing by *H. orphanidis* extracts	Mileski et al., 2016
Aqueous *Tradescantia pallida* extract	*Pseudomonas aeruginosa*	– inhibits both bacterial growth and biofilm formation as well as swarming motility – biofilm treated by *T. pallida* extracts remain premature, likely because of swarming motility inhibition	Kamiya et al., 2019

### Recent Progress and Future Prospects Regarding Mechanism of Action Against Biofilms of Plant Phenolics

*Staphylococcus aureus* biofilms are a major concern for food safety. Interestingly, Wu et al. (2019) observed that coumaroyl-2-hydroxyquinic acid from pine needles of *Cedrus deodara*, not only inhibits *S. aureus* biofilm formation, but also the attachment phase, by inhibiting the transmembrane peptidase sortase A, SrtA. This enzyme catalyzes the covalent binding of surface proteins sharing a typical sorting signal, with a conserved C-terminal LPXTG motif, to cell wall peptidoglycan and these cell wall-anchored proteins initiate bacterial adherence by binding host surface. SrtA inhibitors are thus promising to inhibit the initial stage of biofilm formation. The same group (Liu et al., 2021b) performed combined transcriptomic and proteomic analyses in the absence and in the presence of sub-inhibitory concentrations of coumaroyl-2-hydroxyquinic acid, in order to elucidate its molecular mechanism of action against *S. aureus*. They observed a differential expression of 935 genes and 438 proteins in both situations. Downregulation of surface proteins associated with cell adhesion supports the hypothesis, that this antimicrobial plant phenolic prevents *S. aureus* biofilm formation by inhibiting adhesion of *S. aureus* cells. Bioinformatic analysis also demonstrated that coumaroyl-2-hydroxyquinic acid affects different functions of *S. aureus*, namely at the membrane level.

Recently, when investigating *Adiantum philippense* extract inhibitory activity of biofilm formation by several foodborne pathogens, Adnan et al. (2020) performed an *in silico* molecular docking study of the inhibitory activity of *S. aureus* SrtA by 28 of its identified constitutive phenolics: scutellarin, a glycosyloxyflavone, was found to have the highest affinity for SrtA. Interestingly, this extract also inhibited biofilm formation by other foodborne pathogenic bacteria, and other compounds of the extract had also a high affinity for other adhesins than SrtA. Moreover, this extract significantly inhibited exopolysaccharides production by these bacteria: this is likely another mechanism of biofilm formation inhibition.

Carraro et al. (2014) reported that an olive mill waste extract, inhibiting *E. coli* K12 biofilms formation, also inhibited swarming and swimming motility of these bacteria. Consistently with this observation, they noticed repression of genes for flagellar synthesis and other genes linked to biofilm formation in the presence of this extract. Indeed, reduction of swarming or swimming motility of bacteria by phenolic-rich plant extracts or plant phenolics inhibiting biofilm formation has been frequently reported (e.g., in Table 4: Carraro et al., 2014; Zhang et al., 2014; Vasavi et al., 2015; Yang et al., 2018; Kamiya et al., 2019).

A promising strategy to prevent biofilm formation is based on the identification of quorum sensing inhibitors. Quorum sensing is a mechanism of regulation of gene expression, as a function of microbial cells population density: this mechanism results in the production of extracellular compounds called autoinducers, which alter gene expression of other bacterial cells, when their concentration exceeds a minimal threshold. Since these autoinducers allow bacteria, not only to communicate within species, but also with different species, this mechanism allows bacteria to induce coordinated responses to their environment, like signaling in higher organisms. The phenotypes regulated by quorum sensing include motility, biofilm formation, and resistance to antibiotics (LaSarre and Federle, 2013; Castillo Rivera et al., 2019). Quorum sensing is also involved in the regulation of phenotypes contributing to virulence of bacteria.

Interestingly, several authors have reported the interaction of plant phenolics with homoserine lactones, which are typical auto-inducers of Gram-negative bacteria (Hossain et al., 2017). They reported that methyl gallate had anti-quorum sensing effect on *Chromobacterium violaceum* (an aquatic bacterium used to study the inhibition by diverse molecules of acyl homoserine lactone-dependent quorum sensing, since production of violacein pigment is associated with its quorum-sensing regulated gene expression) and on *P. aeruginosa*, including inhibition of biofilm formation by this bacterium, as well as of its production of exopolysaccharides, which are extracellular polymeric substances in biofilms, and of its swarming motility. Indeed, swarming has been reported to play an important role in the preliminary stage of quorum sensing-regulated bacterial biofilm formation. Since attachment and involvement of quorum sensing are critical steps for the development of biofilms, antimicrobial phenolics acting on these mechanisms are preferred strategies to inhibit biofilm formation.

Baptista et al. (2019) investigated the activity against *E. coli* biofilms of nine phenolics with a catecholic moiety. Increases in the hydrocarbon side chain and lipophilicity, as well as a contribution of hydroxyl groups, were proposed as structural traits favoring anti-biofilm activity of catecholic molecules following a SAR study. SAR and QSAR studies regarding the activity against biofilms of plant phenolics are still scarce. Multiplication of such SAR and QSAR studies should contribute to get a better insight in structural traits of plant phenolics active against biofilms.

Many plant phenolics [e.g., gallic and ferulic acid (Borges et al., 2012), vanillic, caffeic, cinnamic and ferulic acid (Ugurlu et al., 2016), tannic acid (Dong et al., 2018), morin (Sivaranjani et al., 2016), myricetin (Slobodníková et al., 2016), 4-methylcatechol, 4-tert-butylcatechol, and pyrogallol (Baptista et al., 2019), quercetin (Vazquez-Armenta et al., 2020)] and phenolic-rich plant extracts [e.g., garlic extracts (Vadekeetil et al., 2015), *Moringa oleifera* extract (Onsare and Arora, 2015), *Brassicaceae* (radish, radish sprout, red cabbage, kale) extracts (Hu et al., 2019), *Capsicum* peppers extracts (Castillo Rivera et al., 2019), phenolic-enriched extracts produced by enzyme-assisted extraction from oven-dried and lyophilized black grape, apple and yellow pitahaya (Zambrano et al., 2019), tea and turmeric extracts (Tamfu et al., 2020), anthocyanin-rich aqueous extract from purple highland barley bran (Zhang et al., 2020a), avocado peel extract fractions (Trujillo-Mayol et al., 2021)] have been reported to inhibit formation or to eradicate biofilms with undesirable food spoiling or foodborne pathogenic microorganisms.

Interestingly, many authors reported that biofilm formation inhibition by plant phenolics requires lower concentration than to inhibit the growth of the corresponding microorganisms (Carraro et al., 2014; Zhang et al., 2014; Vasavi et al., 2015; Yang et al., 2018; Kamiya et al., 2019). Since biofilms are major causes of cross-contamination of foods, exploitation of antibiofilm activity of plant phenolics is promising to limit foodborne diseases, as well as food spoilage. Disinfection in food processing factories relies upon biocides, requires large amounts of water and generates huge volumes of sewage with high loads of cleaning agents and biocides. In France, 11,000 tons of biocides are used in the agri/food industry per year (data from the French Association of Detergents and Hygiene Product Industry Professionals). Biodegradability of cleaning agents and toxicity of disinfectants are both issues for public authorities, who have established regulations to protect the environment, including the currently implemented EU no 528/2012 Regulation concerning the making available on the market and use of biocidal products in the European Union. However, among cleaning agents and biocides, the most environmentally friendly products are still not widely used. Use of plant phenolics or phenolic-rich plant extracts for this purpose is still in its infancy.

Besides societal and regulatory evolutions related to environmental concerns and the necessity to limit the emergence of multidrug-resistant microorganisms, a broader use of phenolic-rich plant extracts will require, not only a better understanding of the molecular mechanisms leading to biofilm formation prevention or their eradication, but will also benefit from progresses regarding the formulation of delivery systems improving their efficiency. Ongoing progresses in this field are presented in the next section of this review. A promising strategy presented in the next section relies on functionalization of materials with antimicrobial phenolics (e.g., curcumin as proposed by Dogra et al., 2015), in order to prevent biofilm formation on food-contacting surfaces.

## Delivery Modes to Promote the Stability and the Antimicrobial Activity of Plant Phenolics

Antimicrobial plant phenolics direct addition to foods may face the same limits than other antimicrobial compounds, which were listed by Fu et al. (2016) in their review:

− a limited solubility in water, which is a major constituent of perishable foods
− a limited stability, oxidation or heat treatments may affect the activity of some plant phenolics
− uncontrolled release
− alteration of organoleptic properties [alteration of colour or taste (e.g., astrincency of many plant polyphenols)]

Design of food-grade systems to deliver antimicrobial plant phenolics is promising to circumvent these limits. The two main goals of such systems are (i) protection and (ii) sustained release of plant phenolics. Indeed, delivery systems with diverse structures can be prepared with different food-grade components, such as lipids, proteins, carbohydrates, surfactants, and minerals (Zhang et al., 2020b). Controlled release of phenolics from such systems can result from physical entrapment in structures with different geometries (e.g., gels with differing porosity, tortuosity, coated microcapsules …), from weak interactions (hydrophobic or ionic interactions, hydrogen-bonding) between phenolics and delivery systems components or from a mixture of both phenomena. Delivery systems can also be classified according to their dimensions, ranging from molecular inclusion of phenolics in cyclodextrins (Pinho et al., 2015) and the rapidly growing field of nanosystems of delivery [nanoemulsions (Saini et al., 2019; McClements et al., 2021) and nanoparticles (Hu et al., 2017; Hosseini and Jafari, 2020; Vidallon and Teo, 2020; Spizzirri et al., 2021)] to edible coatings or food packagings incorporated with antimicrobial plant phenolics (Mir et al., 2018; Zhu, 2021), and including microemulsions and other microencapsulation systems (Hosseini and Jafari, 2020). Most of systems for controlled delivery of active molecules are issued from the important effort of research for pharmaceutical applications. For controlled delivery of plant phenolics, the goal is often to improve the bioavailability of dietary polyphenols, as reviewed by Hu et al. (2017). Several examples of application to antimicrobial plant phenolics of each of these delivery systems are listed in Table 5.

**Table 5:** Examples illustrating the diversity of systems to deliver antimicrobial plant extracts/phenolics to extend the shelf life or improve the microbial safety of foods or to remove microbial biofilms.

Delivery system component(s)	Antimicrobial plant phenolic/extract	Elaboration method	Antimicrobial activity	References
**Molecular inclusion**				
β-cyclodextrin	Chlorogenic acid	9 h stirring at 50°C followed by drying with a rotary evaporator	– antibacterial activity against *Staphylococcus aureus*, *Bacillus subtilis* and *Escherichia coli* of chlorogenic acid-cyclodextrin complexes was similar, but chlorogenic acid stability against oxidation was improved	Zhao et al., 2010
β-cyclodextrin or 2-hydroxypropyl-β-cyclodextrin	Caffeic acid	Ultrasounds bath for 30 min followed by 24 h at 25°C under constant stirring in the absence of light	– higher *in vitro* antibacterial activity against *Staphylococcus aureus* ATCC 6538 of caffeic acid inclusion complexes with both β-cyclodextrins than caffeic acid alone	Pinho et al., 2015
Methyl-β-D-cyclodextrin	Curcumin	mixing of equal volumes of a 20 mmol·L^−1^ curcumin ethanolic solution and a 20 mmol·L-1 methyl-β-cyclodextrin aqueous solution for 2 h at room temperature followed by ethanol evaporation with a rotary evaporator	– 0.4 mmol·L^−1^ minimal inhibitory concentration against *Escherichia coli* of curcumin-methyl-β-cyclodextrin complexes	Dogra et al., 2015
Cyclodextrin	Propolis	mixing of propolis with cyclodextrin for “green” extraction of phenolic substances, terpenoids, and flavonoids	– the complexes obtained had an antifungal activity suggesting they could be a natural alternative to potassium sorbate	Stagkos-Georgiadis et al., 2021
**Nanoemulsions**				
Aqueous phase with Span^®^ 20 and Tween^®^ 20 emulsifiers to prepare an oil in water (o/w) nanoemulsion	Mangostins extracted from mangosteen peel extract with virgin coconut oil	successive mixing of mangosteen extract in virgin coconut oil and an aqueous phase with 10% (w/v) emulsifiers with an homogenizer and an ultrasonic processor	– nanoemulsions had a higher antibacterial activity against *Escherichia coli* and *Staphylococcus aureus* than the extract (a twice lower minimal inhibitory concentration)	Sungpud et al., 2020
Orange essential oil [70% (v/v)], liquid soya lecithin [20% (v/v)], and cactus pear fruit aqueous extract were mixed to prepare a water in oil (w/o) nanoemulsion	Cactus pear fruit aqueous extract	(w/o) emulsion components were stirred with an ultrasonic processor	– incorporation of 0.4% (v/v) of cactus pear extract (w/o) nanomemulsion in starch film-forming suspensions resulted in films with an *in vitro* antibacterial activity against *Escherichia coli* and *Salmonella typhimurium*	Medina-Pérez et al., 2019
**Nanoencapsulation**				
Chitosan/poly(ethylene oxide; PEO) nanofibers	Pomegranate peel extract	Active chitosan/PEO nanofibers with pomegranate peel extract were prepared by electrospinning of a 4% (w/v) chitosan/PEO blend and 20 g·L^−1^ pomegranate peel extract solution	– addition of active nanofibers in aluminium foil used to wrap raw bovine meat pieces artificially contaminated with *Escherichia coli* O157:H7 resulted in a 2.96 and 5.80 log CFU/g total viable counts reduction compared to meat wrapped in control aluminium foil after 10 days of storage at 4 and 25°C, respectively	Surendhiran et al., 2020
Polycaprolactone (PCL)	Quercetin	Nanoparticles were elaborated by nanoprecipitation followed by freeze-drying [PCL and quercetin acetone solution was added to an aqueous phase with a hydrophilic surfactant (Pluronic F-127) under moderate stirring]. The acetone and water were then removed by vacuum evaporation. The nanosuspension was then ultracentrifuged. The resulting pellets were re-suspended in distilled water and frozen. Frozen nanoparticles were freeze dried.	– quercetin nanoparticles had a higher growth inhibitory effect of *B. subtilis*, *E. coli*, *S. aureus*, and *S. typhimurium* than the same amount of free quercetin for 48 h at 37°C. This difference can be ascribed to sustained release of quercetin from nanoparticles, while free quercetin lost its antibacterial effect against all test bacteria after 2–9 h. The growth of bacteria can then restart.	Dinesh Kumar et al., 2016
Phosphatidylcholine and oleic acid nanoliposomes	Garlic extract	Nanoliposomes were prepared by the thin film hydration method	– garlic extract-loaded nanoliposomes and free garlic extract had a similar *in vitro* antifungal activity against *Penicillium expansum*, *Penicillium herquei*, *Fusarium graminearum*, *Aspergillus flavus*, and *Aspergillus niger* – addition of 0.65 ml of free garlic extract or the same amount of garlic extract-loaded nanoliposomes in bread dough prevented fungal spoilage of sliced bread for 5 days	Pinilla et al., 2019
**Microencapsulation**				
Calcium alginate beads	Onion scale	Ionic gelation by calcium ions of a sodium alginate and onion scale extract solution	– more than 2 log cycle reduction in the growth of *Staphylococcus aureus* and *Pseudomonas fluorescens* in minced chicken meat during chilled storage when 6% (w/v) onion-scale extract encapsulated beads were incorporated	Kanatt et al., 2018
Chitosan and sodium tripolyphosphate	Green tea extract with epigallocatechin gallate as main constituent	Chitosan cross-linked particles loaded with green tea extract were prepared by dropwise addition of a sodium tripolyphosphate solution in a chitosan solution with green tea extract followed by ultrasonication for 3 min	– Total mesophilic aerobic count, coliform bacteria, and yeasts and moulds were monitored for 8 days storage at 4°C of hamburger patties incorporated with green tea extract or green tea-loaded chitosan particles: significant differences in microbial counts in favor of green tea microparticles were observed	Özvural et al., 2016
Maltodextrin, gum arabic	Sugar cane bagasse extract	After solubilization, mixing with a homogenizer and freezing of sugar cane bagasse extract, maltodextrin and gum arabic, microparticles were obtained by freeze-drying	– sugarcane bagasse extract exerted antibacterial activity against *E. coli*, *B. cereus* and *S. aureus* – microencapsulation of sugar bagasse extracts resulted in an improved thermal stability of sugarcane bagasse extract phenolics	Velazquez-Martinez et al., 2021
**Edible coatings or films**				
Sodium alginate	Gallnut ethanolic extract	Preparation of cast films following drying of sodium alginate, gallnut extract and glycerol (as a plasticizer) film forming solutions	– edibles films showed a good *in vitro* antibacterial activity against *Staphylococcus aureus* ATCC 6538 and *Escherichia coli* ATCC 11775	Aloui et al., 2021
Sodium alginate	Pomegranate peel extract (PPE)	PPE or alginate nanospheres containing PPE prepared through water in oil (w/o) emulsification and external gelation with calcium chloride nanoparticles and incorporation in sodium alginate	– fresh chicken breast coated with alginate with PPE nanospheres was less susceptible to microbial growth over 14 days at 4°C than that coated with alginate with “free” PPE – the difference can be ascribed to a more sustained release of PPE antimicrobial compounds from PPE nanospheres	Rahnemoon et al., 2021
Gelatin	*Arbutus unedo* L. fruit methanolic extract	Preparation of films by solvent casting of a gelatin film suspension with glycerol as a plasticizer and *Arbutus unedo* L. fruit extract	– fresh sardine fillets artificially inoculated with *Staphylococcus aureus*, *Listeria monocytogenes*, and *Pseudomonas aeruginosa* were stored at 4°C for 12 days either alone, after covering with *Arbutus unedo* L. fruit extract, or wrapped in gelatin film alone or with *Arbutus unedo* L. fruit extract – interestingly, wrapping of fillets with gelatin-film with *Arbutus unedo* L. fruit extract resulted in the highest reduction of the three inoculated foodborne pathogenic or food spoiling bacterial strains	Bouhanna et al., 2021
Chitosan	Phenolic acids (p-coumaric acid, ferulic acid, gallic acid, vanillic acid, and salicylic acid)	Preparation of chitosan-phenolic acid composite films by solvent casting	Chitosan-ferulic acid composite films better preserved shrimps than other chitosan-phenolic acid films (reduction of total bacterial count and total basic volatile nitrogen over 6 days of storage at 4°C)	Liu et al., 2021a
Chitosan	Cinnamic acids (p-coumaric acid, caffeic acid and ferulic acid)	Carbodiimide-mediated grafting of cinnamic acids with chitosan and subsequent use of grafted chitosan to prepare films by solvent casting	– caffeic acid-grafted chitosan films presenting the highest *in vitro* antibacterial properties were used to wrap pork meat and effectively extended its shelf life to 10 days at 4°C	Yong et al., 2021
**Active packaging**				
Polyethylene terephthalate/polypropylene (PET/PP) films impregnated with olive leaf extract	Olive leaf extract	Supercritical solvent impregnation of PET/PP films with olive leaf extract	Impregnated films inhibited *Staphylococcus aureus*, *Pseudomonas aeruginosa*, and *Escherichia coli* growth and extended cherry tomatoes shelf life by 20 days	Cejudo Bastante et al., 2019
Multilayer polyelectrolyte coating embedding curcumin-cyclodextrin complexes on polyethylene terephthalate (PET) films as a support matrix	Curcumin - carboxymethyl-cyclodextrin complexes	– after a hydrolysis pre-treatment to provide sufficient electric charge to the PET surface, it was electrostatically coated with repeated multilayers comprising alternately deposited positively-charged poly-L-lysine and negatively-charged poly-L-glutamic acid and carboxymethyl-cyclodextrin complexes – carboxymethyl-cyclodextrin molecules were either covalently cross-linked using carbodiimide or left unbound	– coatings with uncrosslinked curcumin carboxymethyl-cyclodextrin complexes were active against *Escherichia coli* both in the dark and when illuminated, while coatings with cross-linked complexes were only active when illuminated due to photodynamic properties of curcumin. – interestingly, cross-linked coatings kept their antibacterial activity following soaking in phosphate buffer saline (PBS) unlike uncrosslinked ones	Shlar et al., 2018
Polyethylene	Pomegranate peel extract	Melt blending of pomegranate peel extract with polyethylene	– 3 days shelf-life extension of pork meat packaged in films with pomegranate peel extract	Hu et al., 2016

### Molecular Inclusion in Cyclodextrins

Molecular inclusion in cyclodextrins of several plant phenolics has increased their solubility in water and their stability before use, while preserving (Zhao et al., 2010) or even in some cases enhancing their antimicrobial activity (Pinho et al., 2015). However, most of antimicrobial activity assays were performed *in vitro* and efficiency of plant phenolics-cyclodextrin complexes antimicrobial activity in foods has still to be demonstrated. This is likely one of the reasons why, to our knowledge, there is still no commercial application of plant phenolics-cyclodextrin complexes to date unlike for natamycin-cyclodextrin complexes for sliced bread preservation in the United States. Progress is also expected to result from a better understanding of the structural and thermodynamic determinants of interactions between phenolics and cyclodextrins: interestingly, Andreadelis et al. (2021) recently investigated the interactions between two antimicrobial phenolics (caffeic acid and rosmarinic acid) and hydroxypropyl-β-cyclodextrin with either 4, or 10 hydroxypropyl moieties by combining experimental methods, such as isothermal calorimetry and calculatory methods, such as molecular dynamics simulation. Further research in this direction should provide data for the rational design of cyclodextrins with optimized structures for the complexation of a given phenolic.

### Nanoemulsion-Based Delivery Systems

Nanoemulsion-based systems to deliver plant antimicrobial compounds in foods have been recently reviewed by McClements et al. (2021). Emulsions are colloidal dispersions of two immiscible fluids. In nanoemulsions, one of them is dispersed in the other one, as small particles with a less than 200 nm diameter. Nanoemulsions can be elaborated with low- or high-energy methods (Saini et al., 2019).

Low-energy methods are based on spontaneous emulsification, phase inversion point, or membrane emulsification. However, they necessitate a high surfactant to oil ratio. Therefore, due to the cost of food-grade surfactants, high-energy methods such as high-pressure homogenization, microfluidization, and sonication are generally preferred for the elaboration of nanoemulsions intended for application in food sector. As illustrated in Table 5, oil-in-water (o/w) nanoemulsions (e.g., antimicrobial phenolics of mangosteen peel soluble in virgin coconut oil; Sungpund et al., 2020) as well as water-in-oil (w/o) nanoemulsions (e.g., water-soluble antimicrobial phenolics of cactus pear; Medina-Pérez et al., 2019) can be prepared and have a protective effect against oxidation of phenolics and/or a positive effect on their antimicrobial activity, by enhancing their dispersion. Although thermodynamically unstable, most of nanoemulsions can be designed to be kinetically stable for sufficient time for commercial applications (McClements et al., 2021), which is advantageous compared to microemulsions. Addition of emulsifiers, which are amphiphilic molecules both facilitating emulsion formation and improving their stability, is necessary. Emulsifiers used can be food ingredients such as proteins, or food additives such as soya lecithin (a phospholipid), modified starch or synthetic food-grade surfactants, such as Tween^®^ 20 [polyoxyethylene (20) sorbitan monolaurate, E 432], which is a non-ionic surfactant.

Besides emulsifiers, the addition of other stabilizers of antimicrobial nanoemulsions, such as thickening agents (e.g., carboxymethyl cellulose, pectin), Ostwald ripening inhibitors (e.g., corn or sunflower oil), weighting agents of the oil phase to inhibit gravitational separation (e.g., dammar gum) is often required to enhance the stability of nano- and micro-emulsions, as briefly reviewed by McClements et al. (2021). As illustrated by Sungpund et al. (2020) in their study on extraction by virgin coconut oil of antimicrobial phenolics from mangosteen peel and subsequent (o/w) nanoemulsion preparation, many other authors explored the possibility to valorize fruits and vegetables wastes through “green” extraction of bioactive compounds, including antimicrobial phenolics and nanoemulsions-based delivery systems, as recently reviewed by Saini et al. (2019). The elaboration of nanoemulsion-based delivery systems is till now better established for essential oils and their components, which are not or poorly soluble in water: essential oil nanoemulsions resulted thus in a better dispersion of their antimicrobial constituents in aqueous systems, often resulting in a higher antimicrobial activity. This approach is more recent for antimicrobial polyphenols and promising for dispersion of poorly soluble in water ones. Future research will not only consider simple (o/w) or (w/o) nanoemulsions of antimicrobial plant phenolics but also the possibilities offered by double (w/o/w) or (o/w/o) nanoemulsions as well as by pickering emulsions to improve their stability and/or their sustained release in food systems. (w_1_/o/w_2_) double emulsions make possible the controlled release of water-soluble compounds trapped within the internal aqueous phase. Pickering emulsions are stabilized by solid colloidal particles. The increased stability of pickering emulsions compared to conventional ones is ascribed to the irreversible adsorption of colloidal particles at the interface and the formation of a thicker rigid layer around the dispersed phase (Sabaghi et al., 2021).

### Nano- or Micro-Encapsulation

Nano- or micro-encapsulation of ingredients extending the shelf life of foods, including some antimicrobial plant phenolics or phenolic-rich plant extracts, have recently been reviewed by Hosseini and Jafari (2020). Nano- or micro-encapsulation of antimicrobial plant phenolics consists in their incorporation into another(other) compound(s), acting as wall material(s) protecting them from factors affecting their stability and/or activity, such as light or oxygen, before and/or during their use and/or controlling their release once used. Most frequently used wall materials are proteins, polysaccharides or their combinations. Their interactions with antimicrobial phenolics, as well as their intrinsic physicochemical properties condition important parameters, such as encapsulation efficiency or kinetics of release of active phenolics. The choice of wall materials also depends on the technique used to encapsulate antimicrobial plant phenolics. Hosseini and Jafari (2020) classified encapsulation techniques in chemical procedures (e.g., interfacial polymerization), physicochemical procedures (e.g., complex coacervation, entrapment in liposomes, ionic gelation of alginate …), physical procedures (e.g., co-extrusion, freeze- or spray-drying …) and emerging encapsulation procedure (e.g., electrospinning/electrospraying).

Several authors compared the *in vitro* and the *in situ* antimicrobial activity of nanoencapsulated antimicrobial plant extracts or phenolics and their free counterparts (Table 5). Interestingly, quercetin-loaded polycaprolactone nanoparticles inhibited for a longer period than free quercetin the *in vitro* growth of foodborne pathogenic bacteria, likely due to its sustained release from nanoparticles (Dinesh Kumar et al., 2016). However, Pinilla et al. (2019) reported that fungal alteration of sliced bread was not better prevented by the addition of garlic extract-loaded nanoliposomes in dough, than by the addition of the same amount of free garlic extract. More recently, Li et al. (2018) prepared chitosan-based nanoparticles simultaneously loaded with catechin and quercetin by ionic gelation of chitosan by sodium tripolyphosphate followed by crosslinking with genipin. The 180 nm size nanoparticles had a significantly lower MIC against *E. coli*, *S. aureus* and *B. subtilis* than catechin, quercetin and chitosan. These lower MIC values might result both from sustained release of catechin and quercetin and from the electrostatic attraction by the negatively charged surface of bacteria of positively charged chitosan nanoparticles.

### Incorporation in Food Contact or Packaging Materials or in Edible Coatings

Incorporation of antimicrobial plant phenolics or extracts in the formulation of food contact materials, deposition of coatings embedding active phenolics or grafting of active phenolics on their surface have been proposed by several authors as a way for controlling microbial growth, cross-contamination, and biofilm formation on food-contacting surfaces (Pei et al., 2014; Dogra et al., 2015; Shlar et al., 2018). Primary food packaging films and trays are also food-contacting surfaces. Moreover, many microbial contaminations of high-added value perishable foods, such as meat pieces or fish fillets, occur in the superficial zone of foods. Therefore, several authors investigated the possibility to functionalize the inner surface of packaging materials with antimicrobial compounds to extend the shelf-life and or improve the microbial safety of perishable foods. Functionalization of food contact materials can rely upon grafting of antimicrobial phenolics resulting from their covalent binding with constitutive polymers or upon their incorporation in the formulation of coatings or packaging materials. In this latter case, coatings or packaging materials act as a reservoir of antimicrobial phenolics, which will reach the surface of food mainly through diffusion. The release of antimicrobial phenolics is controlled by their partition equilibrium between reservoir material and food matrix in direct contact and kinetics of migration of plant phenolics in food contact material and in food, respectively. The release rate of plant phenolics over time should maintain a sufficient concentration to inhibit growth of unwanted microorganisms for a sufficient time to extend the shelf life of food in contact.

In the European Union, the possibility to launch active food packaging systems is authorized since 2009 [Regulation (EC) no 450/2009, 2009]. However, only molecules with a food additive or a food ingredient status might be released from such food packaging systems to foods. This could be limiting for the commercial application of packaging materials releasing phenolic-rich antimicrobial plant extract, since not all molecules transferred from film to food would be characterized. The use of pure phenolics with a food additive [e.g., curcumin (E 100) anthocyanins (E 163) as colorants] or a food ingredient status (e.g., naringin, tannic acid, or hesperetin as food flavourings) would thus be preferred.

Up to now, most of food packaging materials are plastic-based materials made of polyolefins, such as polyethylene (PE), polyethylene terephthalate (PET) or polypropylene (PP) and are elaborated by extrusion-injection for rigid materials such as trays or by extrusion-blowing for films. If antimicrobial compounds are incorporated in their formulation before their elaboration, this requires that antimicrobial activity of phenolics is not lost due to the high temperature conditions (which can exceed 200°C for the extrusion of polypropylene for instance) and the shear stress prevailing during extrusion process. Interestingly, Hu et al. (2016) blended 15 g·kg^−1^ of pomegranate peel extract with PE by twin-screw extrusion at 160–190°C to elaborate active films and reported a decrease in total volatile basic nitrogen (TVB-N) during refrigerated storage of pork meat packaged in such film and an extension of 3 days of its shelf life. This suggests that pomegranate peel extract kept its antimicrobial activity following extrusion of films. Similarly, Cottaz et al. (2019) incorporated a synthetic antimicrobial phenolic, isobutyl-4-hydroxybenzoate in poly(ethylene-co-vinyl acetate; EVA), linear low density polyethylene (LLDPE), and PP by melt-blending to prepare pellets, which were subsequently used to prepare heat-pressed films: all films had an antibacterial activity, demonstrating thus the preservation of isobutyl-4-hydroxybenzoate antibacterial activity after melt processing. Antimicrobial polyphenols, which are less volatile than antimicrobial essential oil components, and which are not denatured by temperatures exceeding 140–150°C like antimicrobial proteins, such as lysozyme, or inactivated by heat, such as antimicrobial peptides like nisin, are thus promising compounds for incorporation in packaging materials elaborated by extrusion.

However, for regulatory reasons, it might be more interesting to incorporate food-grade phenolic-rich extracts of plants in edible films or coatings: the only condition is that their formulation only contains food ingredients, additives or processing aids. When developing active packaging films or edible films or coatings releasing antimicrobial plant phenolics in the superficial zone of foods (e.g., Zam and Ali, 2018), one must keep in mind that such delivery systems are advantageous, only if a more sustained release of phenolics over time is observed, compared to dipping foods in a plant phenolic solution or spraying a plant phenolic solution on their surface before packaging. Interestingly, Bouhanna et al. (2021) reported that the growth of bacteria contaminating the surface of sardine fillets was better inhibited, when fillets were wrapped with a gelatin film incorporated with *Arbutus unedo* L. fruit extract, than when they were covered with a layer of *A. unedo* L. fruit extract. This difference is likely due to the sustained release of *A. unedo* L. fruit extract antibacterial constituents, resulting from their entrapment in a gelatin-based network and/or to the weak interactions between gelatin and these constituents. Recently, Rahnemoon et al. (2021) compared the preservation of fresh chicken breast meat in alginate coatings with either free pomegranate peel extract or alginate nanospheres loaded with pomegranate peel extract: fresh chicken meat had a longer shelf life when coated with pomegranate peel extract nanospheres. This difference was attributed to the more sustained release of active phenolics from pomegranate peel extract nanospheres. Protein and polysaccharides are the most frequently used biopolymers for the preparation of edible coatings or films. Interestingly, some polysaccharides, such as chitosan, have an intrinsic antimicrobial activity, which can act synergistically with antimicrobial plant phenolics. Yong et al. (2021) recently grafted cinnamic acids with chitosan and observed that films elaborated with caffeic acid-chitosan conjugates had the highest antimicrobial activity. Many other authors recently investigated the effect of the addition of polyphenols to polysaccharides for food packaging applications, as recently reviewed by Zhu (2021).

Further commercial development of antimicrobial plant phenolics delivery systems requires that more studies include their application to a real perishable food to evaluate their potential for shelf life extension. Moreover, there is still a too limited number of studies including an application of such controlled delivery systems to real foods, without including a comparison with the effect of direct addition of the same amount of antimicrobial plant phenolic or extract, which is necessary to estimate the added-value of controlled delivery systems, from which formulation and elaboration represent an additional cost. As suggested by McClements (2018) in his review proposing a “delivery by design” approach for the design of efficacious nanoparticle- and microparticle-based systems to deliver active agents, the development of such systems has to be inspired by quality by design approach used in industrial sector.

## Conclusion and Perspectives

Compared to the diversity of antimicrobial plant phenolics and phenolic-rich plant extracts and the current use of biocides for disinfection of food production facilities and of food preservatives, their application is still in its infancy. However, more and more formulations containing phenolic-rich plant extracts with antimicrobial activity are proposed. For instance, the interested reader can refer to Sima et al. (2018), who reported that a commercial formulation with namely citrus, grape seed and oregano extracts in addition to lactic and citric acids effectively reduced *in vitro* and *in vivo* pathogenicity of T6SS positive *Campylobacter jejuni* and *Campylobacter coli* chicken isolates. T6SS positive highly virulent *Campylobacter* spp. are positive for the Type VI secretion system (T6SS), which have an increased ability to invade the host gastrointestinal epithelium are highly prevalent in poultry. Therefore, their observations are of particular importance, since preventing *Campylobacter* spp. infections in humans is considered a public health priority.

Research on antimicrobial activity of edible phenolic-rich plant extracts or plant phenolics expands rapidly, not only benefitting from progress in -omics to better describe their molecular mechanisms of action, but is also being stimulated by (i) the “clean label” marketing trend, which stimulates the search of natural ingredients, as alternatives to synthetic food preservatives, (ii) the necessity to develop more sustainable food systems, favoring the valorization of antimicrobial phenolics-rich food by-products or wastes, by following the principles of circular economy, (iii) the environmental and microbial safety considerations to limit the emergence of multi-drug resistant microorganisms, and (iv) the search for alternatives to plastic food packagings [as illustrated by the “single use plastics” directive in the European Union (EU 2019/904 Directive, 2019)]. However, despite this scientific and societal context favorable to the expansion of the use of antimicrobial phenolic-rich plant extracts or plant phenolics in the food sector, present limitations, such as (i) high cost for extraction of antimicrobial plant phenolics from plants resulting from climate variability and long growth cycle of plants, the requirement of solvents and/or high energy consuming extraction methods or (ii) the necessity to perform more in-depth studies regarding the toxicity of some plant phenolics, which can be pro-oxidant or mutagenic at high dosage and to check that their broad use will not promote the emergence of resistant microbial strains. Some of these challenges were recently reviewed and discussed by Ofosu et al. (2020). The issue of the cost can partly be addressed by applying hurdle technology principles to combine antimicrobial plant phenolics application with other antimicrobial factors (processes, antimicrobial compounds) acting synergistically.

## Author Contributions

NO and PD conceptualized, searched initial bibliography, wrote the first manuscript draft, and revised the manuscript. All authors have read and approved the final version of the manuscript.

## Funding

The authors gratefully acknowledge the French National Agency for Research (ANR-14-CE20-0005-01 ACTIPHEN) for the financial support of a project on the applicability of antimicrobial plant phenolics for food preservation, which contributed to five the authors more practical insight in the subject of give this review.

## Conflict of Interest

The authors declare that the research was conducted in the absence of any commercial or financial relationships that could be construed as a potential conflict of interest.

## Publisher’s Note

All claims expressed in this article are solely those of the authors and do not necessarily represent those of their affiliated organizations, or those of the publisher, the editors and the reviewers. Any product that may be evaluated in this article, or claim that may be made by its manufacturer, is not guaranteed or endorsed by the publisher.
